# Capillary Electrophoresis With Amperometric Detection for Neurotransmitter Analysis: Principles, Electrode Materials, Methodologies, and Applications

**DOI:** 10.1002/elps.70099

**Published:** 2026-05-02

**Authors:** Petr Kubáň, Jiří Volánek, Nguyen Thi Thu Trang, Tran Dai Lam

**Affiliations:** ^1^ Department of Bioanalytical Instrumentation Institute of Analytical Chemistry of the Czech Academy of Sciences Brno Czech Republic; ^2^ Department of Chemistry, Faculty of Science Masaryk University Brno Czech Republic; ^3^ Institute of Materials Science Vietnam Academy of Science and Technology Ha Noi Vietnam

**Keywords:** amperometric detection, biological fluids, capillary electrophoresis, electrodes, neurotransmitters

## Abstract

Capillary electrophoresis with amperometric detection has become a powerful tool for the determination of electroactive neurotransmitters due to its high separation efficiency, minimal sample consumption, and excellent detection sensitivity. This review provides the first comprehensive summary specifically on monoamine neurotransmitters and related compounds in biological systems. Fundamental principles of amperometric detection, electrode positioning strategies, and approaches to high‐voltage decoupling are described, followed by a critical comparison of electrode materials including carbon fiber, glassy carbon, graphite, noble metals, boron‐doped diamond, and chemically modified or multielectrode designs. Their impact on noise, sensitivity, fouling resistance, and ease of integration is highlighted. Particular attention is given to background electrolyte composition, where phosphate buffers are most widely used, while 2‐(*N*‐morpholino)ethanesulfonic acid systems have enabled the lowest limits of detection, often reaching the attomole range. Applications span human urine, blood, lymphocytes, brain tissue, microdialysates, invertebrate tissues, and single cells, demonstrating capillary electrophoresis with amperometric detection as a versatile platform for neurochemical analysis. Advances in online preconcentration, dynamic pH junctions, and redox‐cycling electrodes have further improved selectivity and sensitivity. Finally, current limitations, such as electrode fouling and interface robustness, are discussed together with emerging trends, including clinical translation and in vivo neuroanalysis.

## Graphical abstract

This review provides a comprehensive summary of monoamine neurotransmitter analysis by capillary electrophoresis with amperometric detection. Fundamental principles of amperometric detection, electrode positioning strategies, and approaches to high‐voltage decoupling are described, followed by a critical comparison of electrode materials, selection of separation electrolytes, and applications to biofluid samples.

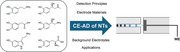

**Abbreviations**
3‐MT: 3‐methoxytyramine
5‐HIAA: 5‐hydroxyindoleacetic acid
5‐HT: serotonin (5‐hydroxytryptamine)
AA: ascorbic acid
AD: amperometric detection
BDD: boron‐doped diamond
BGE: background electrolyte
CA: cellulose acetate
CAPS: 3‐(cyclohexylamino)‐1‐propanesulfonic acid
CCE: carbon composite electrode
CE: capillary electrophoresis
CE–AD: capillary electrophoresis with amperometric detection
CE–ED: capillary electrophoresis with electrochemical detection
CFE: carbon fiber electrode
CMCE: chemically modified carbon electrode
CNS: central nervous system
CNT: carbon nanotube
CNTPE: carbon nanotube paste electrode
CPE: carbon paste electrode
CSF: cerebrospinal fluid
CVD: chemical vapor deposition
CZE: capillary zone electrophoresis
DA: dopamine
DHBA: 3,4‐dihydroxybenzylamine
DOPAC: 3,4‐dihydroxyphenylacetic acid
E: epinephrine (adrenaline)
EOF: electroosmotic flow
FASS: field amplified sample stacking
FSCV: fast‐scan cyclic voltammetry
GCE: glassy carbon electrode
Him: histamine
HV: high voltage
HVA: homovanillic acid
id: inner diameter
IDA: interdigitated array
IDRA: interdigitated ring array
L‐DOPA: 3,4‐dihydroxy‐L‐phenylalanine
LDME: levodopa methyl ester
LOD: limit of detection
MCE: microchip electrophoresis
MES: 2‐(*N*‐morpholino)ethanesulfonic acid
MEKC: micellar electrokinetic chromatography
MS: multiple sclerosis
MNTs: monoamine neurotransmitters
MT: melatonin
MWCNT: multiwalled carbon nanotube
na5‐HT: *N*‐acetyl‐5‐hydroxytryptamine
naDA: *N*‐acetyldopamine
naOA: *N*‐acetyloctopamine
naTA: *N*‐acetyltyramine
NE: norepinephrine (noradrenaline)
NTs: neurotransmitters
OA: octopamine
OCE: on‐capillary electrode
PC12: pheochromocytoma cell line
PD: Parkinson's disease
Pdop: polydopamine
PK: pharmacokinetics
SCE: saturated calomel electrode
SDS: sodium dodecyl sulfate
SPE: solid phase extraction
S/N: signal to noise
TA: tyramine
TES: 2‐[(2‐hydroxy‐1,1‐bis(hydroxymethyl)ethyl)amino]ethanesulfonic acid
TrA: tryptamine
VMA: vanillylmandelic acid

## 1 Introduction

Capillary electrophoresis (CE) coupled with electrochemical and notably amperometric detection (AD) has become a valuable analytical tool for the determination of electroactive compounds. AD is well accepted for its ability to provide the picomolar sensitivity for analytes that are easily oxidized or reduced and offers selectivity often complementary to that obtained with optical detection techniques. Already in the pioneering article on CE coupled to AD by Wallingford and Ewing [1], the detection of monoamine neurotransmitters (MNTs) was shown as a model example. Neurotransmitters (NTs) constitute a chemically heterogeneous group of analytes that are typically present at low concentrations and often coexist with numerous potential interferents in biological samples. Their accurate detection and quantification are essential for elucidating neurochemical pathways, understanding the molecular basis of neurological disorders, and developing diagnostic and therapeutic strategies. CE coupled with AD is particularly well‐suited for such analyses. CE–AD has been reviewed previously, focusing mainly on instrumental aspects, particularly the integration of AD with the high‐voltage part of the CE system. Table 1 provides a comprehensive summary of the review articles published to date, summarizing the focus and main objectives of each contribution. Table 1 is organized into three sections according to review type. The first section comprises reviews dedicated to CE–AD [2, 3, 4, 5, 6, 7], the second section includes review articles addressing amperometric detection in microchip electrophoresis [8, 9, 10, 11], and the third section covers application‐oriented reviews [12, 13, 14, 15, 16]. In general, CE coupled to AD is a cost‐effective and robust approach when low‐volume samples and high‐sensitivity measurements of MNTs are required and complement the UV–vis, fluorescence, and mass spectrometric detection. To date, no comprehensive review has been devoted exclusively to the analysis of NTs by CE–AD; thus, the present article is intended to address this gap by providing a systematic overview of CE–AD applications in NT analysis. Specifically, we shall summarize the various electrode materials and detection strategies that have been developed, the instrumental arrangements optimized for these measurements, and the background electrolyte (BGE) compositions tailored for NT separations. In addition, we shall highlight representative applications in neuroscience research and conclude with a perspective on future developments and potential directions for advancing CE–AD as a robust tool in neurochemical investigations.

**TABLE 1:** Summary of previous review articles on CE–AD and related topics.

Publication year	First author surname	Review details	Reference
1997	Voegel	First comprehensive review; detailed coverage of capillary/electrode decoupling strategies and electrode positioning	[2]
1998	Holland	Advantages of CE–ED over the preceding 3 years, amperometry as the most widely adopted detection mode	[3]
1998	Kappes	Review of amperometric, potentiometric, and conductometric detection in CE systems	[4]
1998	Matysik	Review of end‑column designs without the use of an electric field decoupler	[5]
2000	Baldwin	Sequel to the previous review (1997), emphasis on decoupling, electrode geometries, dual‑electrode systems, and emergence of microfabricated CE–ED devices	[6]
2002	Holland	Explicit comparison of amperometric and voltammetric detection; predominance of oxidative amperometry; summarized decoupling strategies and electrode designs	[7]
2004	Vandaveer	Review of the integration of amperometric detection with microchip electrophoresis (MCE)	[8]
2015	Saylor	Review of coupling of microdialysis with MCE for near‑real‑time neurochemical monitoring	[9]
2020	Schilly	Summary of biological applications of MCE–AD	[10]
2021	Costa	Comprehensive review of MCE–electrochemical device designs and materials	[11]
2005	Powell	Review of applications of CE to neuroscience, with respect to various biological samples and single‐cell analysis	[12]
2006	Tsunoda	Separation methods (CE, LC, GC) used for analysis of catecholamines with different detection modes are reviewed	[13]
2008	Lapainis	Review of CE in neuroscience with particular attention to detection and sampling	[14]
2009	Perry	General review on various analytical techniques for neurotransmitter analysis, including CE	[15]
2015	Sanchez‐Lopez	Review on CE contribution to neuroscience between 2008 and 2014	[16]

## 2 Neurotransmitters and Their Importance

NTs are endogenous chemical messengers responsible for transferring signals between neurons, muscle cells, and glandular cells, thereby ensuring brain–body communication and systemic homeostasis. The first NT, acetylcholine, was discovered in 1921 by Otto Loewi in his classical frog heart experiment, a finding that later earned him the Nobel Prize [17]. Since then, more than one hundred distinct NTs have been identified [18], highlighting the chemical and functional diversity of neuronal signaling. Classification is often based on chemical structure, and major categories include amino acids (e.g., glutamate, γ‐aminobutyric acid), biogenic amines (e.g., dopamine [DA], norepinephrine [NE], epinephrine [E], serotonin [5‐HT], histamine [Him]) and their primary precursors/metabolites (3,4‐dihydroxy‐L‐phenylalanine (L‐DOPA) and 3,4‐dihydroxyphenylacetic acid (DOPAC), soluble gases (e.g., nitric oxide, hydrogen sulfide), and other NTs, such as acetylcholine and choline [19].

This structural diversity underpins their functional complexity. NTs regulate virtually every aspect of brain and body physiology: they modulate cognition, learning, mood, sleep, appetite, memory, and consciousness, but also cardiovascular, renal, hormonal, and immune functions. Because of these wide‐ranging roles, changes in NT concentrations in the central nervous system are strongly associated with psychotic disorders such as schizophrenia and depression, neurodegenerative diseases such as Parkinson's, Alzheimer's, Huntington's disease, and epilepsy, as well as various systemic conditions including arrhythmias, thyroid dysfunction, glaucoma, and inflammatory syndromes [20]. MNTs are not only essential for normal neurobiology but also represent key biomarkers and therapeutic targets in clinical neuroscience, and this is why this review is devoted to these compounds. In addition, their chemical structure and resulting electroactive properties (catechol or indole function) make them ideally suited for direct amperometric detection without prior derivatization or specific reaction with modified electrode surface, as would be needed in other classes of neurotransmitters.

Additional electroactive metabolites and degradation products of monoamine neurotransmitters, such as 5‐hydroxyindoleacetic acid (5‐HIAA), homovanillic acid (HVA), and 3‐methoxytyramine (3‐MT), are also analytically and biologically relevant. However, they show significant structural differences that alter their electron‐transfer kinetics, oxidation potentials, and adsorption behavior at electrode surfaces. Their determination by CE–AD has been reported more sporadically and with greater variability across studies.

Thus, the analytes included in this review are DA, NE, E, 5‐HT, Him, L‐DOPA, and DOPAC. These NTs are highly conserved in evolution and are distributed throughout the central and peripheral nervous systems, where they coordinate critical functions such as cognition, arousal, stress responses, cardiovascular regulation, and metabolic control [15, 21, 22, 23]. The chemical structures and important physicochemical characteristics of the MNTs considered herein are shown in Table 2, including also a brief description of their physiological functions and relation to possible disease diagnostics.

**TABLE 2:** Physicochemical and functional properties of selected neurotransmitters and related compounds.

Compound (abbreviation)	Chemical structure	Formal oxidation potential E^0^ (V vs. Ag/AgCl)^a^	pKa (values)^a^	Principal function(s) in neuroscience with reference to articles
Dopamine (DA)	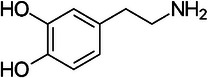	+0.11 V	pKa_1_ ≈ 9.3 (amino), pKa_2_ ≈ 10 (phenolic OH)	Regulates motor control, motivation, reward, and executive functions, and its dysregulation is strongly linked to Parkinson's disease, schizophrenia, and addiction [15, 24, 25, 26]
Norepinephrine (NE)	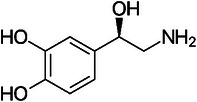	+0.40 V	pKa_1_ ≈ 8.9 (amino), pKa_2_ ≈ 9.5 (phenolic OH)	Mediates attention, vigilance, stress responses, and memory consolidation [27], with imbalances associated with Parkinson's disease, depression, and ADHD [28]
Epinephrine (E)	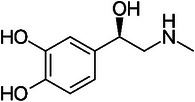	+0.24 V	pKa_1_ ≈ 8.9, pKa_2_ ≈ 9.7	Neurotransmitter and hormone driving the “fight‐or‐flight” response, leading to increased heart rate, bronchodilation, and enhanced energy metabolism [27, 29]. Abnormal signaling contributes to stress‐related and anxiety disorders [30]
Serotonin (5‐HT)	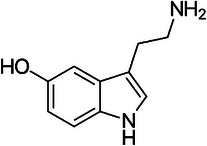	+0.40 V	pKa_1_ ≈ 9.9 (amino), pKa_2_ ≈ 10.1 (indole NH)	Regulates mood, appetite, sleep, and pain, and its dysregulation is implicated in depression, anxiety, OCD, autism spectrum disorders, and substance abuse [31, 32, 33]
Histamine (Him)	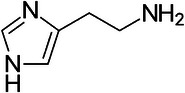	+1.1 V	pKa_1_ ≈ 9.8 (amino), pKa_2_ ≈ 5.8 (imidazole)	Histamine contributes to arousal, cognition, and endocrine and immune regulation, with altered signaling associated with Alzheimer's disease, Parkinson's disease, schizophrenia, and vascular dementia [34, 35]
DOPAC	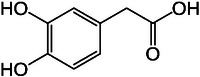	+0.15 to +0.20 V	pKa_1_ ≈ 4.3 (COOH), pKa_2_ ≈ 9.5 (phenolic OH)	Major oxidative metabolite of DA, serves as an indicator of dopamine turnover and monoamine oxidase activity in neurochemical studies
L‐DOPA	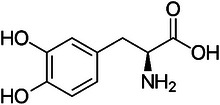	+0.55 V	pKa_1_ ≈ 2.3 (COOH), pKa_2_ ≈ 9.7 (NH_3_^+^), pKa_3_ ≈ 10.3 (phenolic OH)	Immediate metabolic precursor of DA, used therapeutically to restore dopaminergic transmission in Parkinson's disease

^a^For details and references, see Table S1 in the Supporting Information.

## 3 Capillary Electrophoresis With Amperometric Detection in Neurotransmitter Analysis

CE is a liquid‐phase separation technique in which analytes are separated according to differences in their electrophoretic mobility, which depends on the charge‐to‐size ratio, under the influence of a high electric field. Separations are typically performed in fused‐silica capillaries with an inner diameter (id) of 25–75 µm and lengths of several tens of centimeters, operated at separation voltages up to 30 kV [36]. The use of high electric fields in combination with small capillary diameters enables rapid separations with very high efficiency, often exceeding hundreds of thousands of theoretical plates.

In uncoated fused‐silica capillaries, separation is strongly influenced by the electroosmotic flow (EOF), which arises from the negatively charged silanol groups at the capillary wall. Under an applied electric field, the resulting bulk flow of the electrolyte transports all charged and neutral species toward the detector, allowing simultaneous analysis of a wide range of analytes [37]. The direction and magnitude of EOF, and thus migration order and analysis time, can be controlled by buffer composition, pH, ionic strength, or surface modification of the capillary. Importantly, these parameters also influence the ionization state of the analytes themselves, for example, through protonation or deprotonation, thereby altering their effective charge and electrophoretic mobility. This dual control over both bulk flow and analyte properties provides exceptional flexibility in tuning selectivity and resolution in capillary electrophoresis [36, 37].

CE is inherently suited for the analysis of very small sample volumes; injected samples usually represent only 1%–2% of the total capillary volume, corresponding to nanoliter‐scale amounts [38]. This low sample requirement, combined with high separation efficiency and short analysis times, is particularly advantageous for neurochemical applications involving limited sample availability, such as single‐cell analysis, brain microdialysates, or other microscale biological samples.

Capillary inner diameter plays an important role in both separation performance and detection compatibility. In contrast to optical detection techniques, where sensitivity decreases markedly with reduced optical path length, electrochemical detection is largely independent of capillary id [4]. On the contrary, the use of smaller ids is often advantageous for AD. Smaller ids result in increased capillary electrical resistance, which leads to lower separation currents and reduced Joule heating at a given applied voltage. This reduction in thermal effects improves separation stability and facilitates reliable electrical decoupling between the high‐voltage separation circuit and the electrochemical detector, thereby enabling stable and low‐noise amperometric measurements [39].

When coupled with amperometric detection (CE–AD), CE offers a combination of high separation efficiency, sensitive and selective detection for redox‐active neurotransmitters. Monoamines, catecholamines, indoleamines, and related metabolites can be resolved within short electrophoretic windows and detected directly without derivatization. CE–AD further benefits straightforward integration with on‐line sampling approaches such as microdialysis [9]. Sensitivity can be enhanced by incorporating electrophoretic preconcentration strategies, including field‐amplified stacking, dynamic pH junctions, or transient isotachophoresis, often achieving sub‐nanomolar detection limits.

Despite these advantages, several technical challenges limit the broader adoption of CE–AD. A fundamental issue is the electrical incompatibility between the high‐voltage separation field and the low‐current amperometric detector, necessitating effective decoupling of the separation voltage from the working electrode. Off‐column decoupling strategies, such as porous glass joints [1] or ion‐exchange membranes [2], add mechanical complexity and may introduce band broadening or analyte loss. End‐column detection avoids external decouplers but typically requires very small capillary inner diameters or low‐conductivity background electrolytes to suppress separation currents [5]. Background electrolyte (BGE) composition strongly influences performance, as biologically relevant buffers such as phosphate increase ionic strength and background currents, whereas lower‐conductivity buffers improve electrochemical sensitivity but deviate from physiological conditions.

Electrode fouling represents another major limitation, especially for catecholamines and complex biological matrices. Oxidative by‐products of NTs can undergo further chemical reactions, forming polymeric or strongly adsorbing species that passivate the electrode surface and lead to progressive signal attenuation and reduced reproducibility. This phenomenon has been experimentally characterized for neurotransmitters, where repeated injections resulted in gradual sensitivity loss due to surface contamination [40]. To mitigate these effects, pulsed amperometric waveforms incorporating cleaning and reactivation steps have been introduced to electrochemically regenerate the electrode surface during analysis [40, 41]. In addition, the use of alternative electrode materials and surface modifications can significantly improve operational stability. For instance, permselective or polymer‐modified electrodes (e.g., Nafion coatings or melanin‐type films) have been employed to reduce adsorption of interfering species and improve long‐term performance during catecholamine analysis [42, 43]. Nevertheless, even with these strategies, maintaining long‐term stability in highly complex biological matrices remains challenging.

Overall, CE–AD combines exceptional separation efficiency, minimal sample consumption, and high sensitivity for electroactive neurotransmitters, making it a powerful tool for fundamental neurochemical studies and microscale analysis. Challenges related to voltage decoupling, electrode fouling, mechanical robustness, and matrix effects continue to hinder widespread implementation. Ongoing developments in electrode materials, capillary and chip‐based architectures, and decoupler‐free detection strategies are expected to further improve the reliability and applicability of CE–AD in neurotransmitter research, as will be discussed later here.

## 4 Principle of Amperometric Detection and its use in Neurotransmitter Analysis

AD in CE relies on the electrochemical oxidation or reduction of analytes at a working electrode held at a constant potential relative to a reference electrode (and auxiliary electrode in some cases). When an electroactive substance migrates to the electrode surface, electron transfer occurs and generates a faradaic current proportional to analyte concentration [7]. This current is measured typically with a potentiostat, enabling high‐sensitivity detection for redox‐active species such as catecholamines, indoleamines, and certain amino acids, to name a few examples. The technique is inherently well‐suited to CE because miniaturized electrodes, often carbon fibers (typical diameter is between 5 and 33 µm) or noble metal wires (typical diameter is between 10 and 200 µm), match the dimensions of separation capillaries and provide sensitive detection. This is because the reduction in electrode size and thus corresponding surface area markedly decreases nonfaradaic (capacitive) charging currents and background noise. While the absolute signal decreases as well with electrode miniaturization, the noise decreases at least proportionally, leading to preserved or even improved signal‐to‐noise (S/N) ratios compared with larger electrodes [44]. In contrast to optical methods, the concentration sensitivity of AD is not compromised by the id of the separation capillary. Not surprisingly, separation and AD detection of NTs has been achieved even in sub‐micrometer id capillaries [45, 46], while the use of other detection techniques in such capillaries would be extremely challenging.

All MNTs can be detected amperometrically because they are electroactive, which is due to their chemical structures. Oxidation potentials are compiled in Table 2 and Table S1, and the associated oxidation reactions are provided in Table S3. The primary chemical structure responsible for the easy oxidation of DA, NE, E, L‐DOPA, and DOPAC is the ortho‐dihydroxybenzene (catechol) group, which undergoes a two‐electron oxidation to its corresponding quinone at the electrode surface [47]. 5‐HT is an indoleamine with a 5‐hydroxybenzene group that is also easily oxidizable [48]. On the other hand, the imidazole ring of Him is only weakly electroactive, and its oxidation requires relatively high potentials (≥+1.1 V vs. Ag/AgCl) [49] or modified electrode surfaces that catalyze or mediate the oxidation of the imidazole moiety can be employed to enable sensitive detection [50].

The LODs achieved with AD are typically in the nanomolar to low micromolar range, appropriate for physiological concentrations of various compounds in brain tissue, microdialysis samples, or single cells. Selectivity is tunable by adjusting the applied detection potential, allowing discrimination among compounds with different oxidation potentials. The following sections describe the various strategies for coupling AD to CE, with particular emphasis on approaches designed to electrically decouple the high separation voltage and associated currents from the sensitive amperometric measurement electronics.

### 4.1 Decoupling Strategies

The high separation voltages applied in CE (10–30 kV) generate high background currents on the order of units to tens of microamperes that can interfere with the faradaic current from the amperometric detector that is typically on the order of picoamperes [2, 4]. If not adequately isolated, these separation currents can introduce additional electrical noise at the working electrode, thereby degrading the S/N ratio [51]. Lu and Cassidy [39] concluded that the detector background noise increased significantly for capillaries with an id greater than 25 µm without proper decoupling.

In addition, improper decoupling may allow part of the separation current to be unintentionally grounded through the detection electronics, potentially compromising both detector stability and electrical safety [1, 52]. A further consequence is the possibility of a potential shift at the working electrode [53], such that the effective electrode potential differs from the nominally applied value, leading to distorted voltammetric behavior and reduced selectivity. These considerations form the fundamental motivation for the various decoupling strategies that have been developed, all of which aim to electrically isolate the high‐voltage separation circuit from the low‐current detection circuitry while preserving separation efficiency and analytical sensitivity.

**FIGURE 1:**
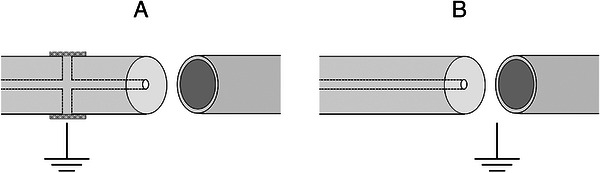
Schematics of detection approaches in CE–AD: (A) off‐column detection (with decoupling), facilitated by a fracture covered with porous material (e.g., Nafion, cellulose acetate, porous glass); (B) end‐column detection (without decoupling).

The most applied electrical decoupling approach is the so‐called **off‐column** (i) detector arrangement (Section 4.1.1, hereafter, Figure 1A) [2, 6, 7]. Although this approach yields low noise of the measured signal, it is difficult to construct and can result in possible analyte loss and band broadening. Thus, the other option is the use of the so‐called **end‐column** (ii) detector arrangement that relies on the small id of the separation capillary or, in some cases, on the use of low conductivity separation electrolytes (Section 4.1.2), see for instance [5]. Figure 1 shows the fundamental difference between off‐ and end‐column detector arrangements. Both detection arrangements are described in detail below.

#### 4.1.1 Off‐Column Detection

The off‐column configuration is characterized by electrical decoupling between the separation capillary and the detection capillary—these may be two capillary fragments [1, 54] or a single capillary modified by introducing a controlled fracture [55] (see Figure 1A). The outlet/fractured separation capillary is typically immersed in an electrolyte reservoir containing the grounding or high‐voltage (HV) electrode of the CE system, where the separation voltage and current effectively drop across the capillary end [2]. The downstream working and reference AD electrodes are positioned at the outlet of the detection capillary and are thus electrically isolated from the separation field. During analysis, analytes from the sample migrate through the separation capillary due to the combined effects of electrophoretic mobility and electroosmotic flow (EOF). After electrical decoupling, analyte transport to the working electrode is driven by electroosmotic flow generated in the upstream separation capillary, which continues as pressure‐driven plug flow through the short detection capillary despite the absence of an axial electric field. To minimize band broadening and loss of temporal resolution, the length of the detection capillary must be kept very short (often in the millimeter range) [7]. In the first report, Wallingford and Ewing employed a porous‐glass sleeve to connect two precisely aligned capillary ends, thereby enabling electrical grounding while maintaining hydrodynamic continuity [1]. However, due to the demanding fabrication of such sleeves, a range of simpler and more practical decoupler designs was subsequently introduced. One widely adopted approach involved creating a controlled fracture in the capillary wall [2] that is coated with Nafion [56] or cellulose acetate (CA) [55, 57] to suppress analyte loss and maintain ionic conductivity at the decoupling interface. Alternatively, decoupling junctions were fabricated by etching a small opening in the capillary wall using hydrofluoric acid, followed by sealing with epoxy adhesive to ensure mechanical stability [58]. Beyond these designs, a variety of materials have been explored for decoupler construction, including porous graphite tube [59], palladium tube [60], and Teflon [61].

#### 4.1.2 End‐Column Detection

The end‐column configuration (see Figure 1B), first introduced by Huang et al. [62], exploits the fact that when narrow‐bore capillaries are used, noise originating from the CE high voltage is largely suppressed. This is a consequence of the high electrical resistance of small‐diameter capillaries, which causes a rapid potential drop at the capillary outlet. Typically, capillaries with id below 25 µm are used to achieve a practical balance between operational robustness (flushing, bubble formation, and clogging) and amperometric detection performance [39, 63]. The use of capillaries with id below 10 µm, or even in the sub‐micrometer range, has also been reported [45, 46]. Conversely, larger‐bore capillaries (50–75 µm id) may be used in end‐column detection only under low‐conductivity conditions, though they generally exhibit higher LODs [5].

### 4.2 Capillary/Electrode Alignment

**FIGURE 2:**
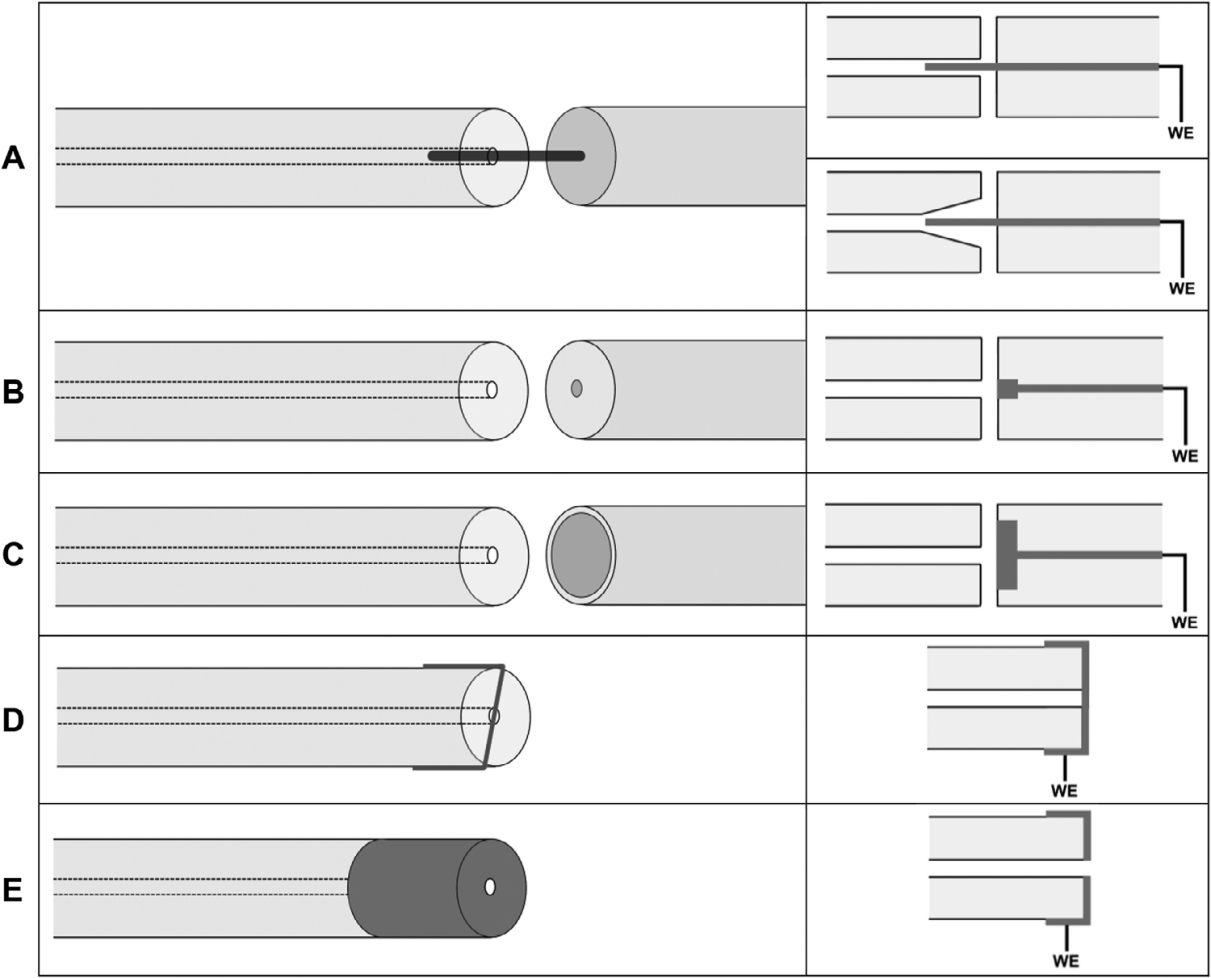
The schematic representation of different separation capillary to electrode configurations. (A) in‐capillary detection (right‐hand side shows side view of a normal and etched capillary ends), (B) wall jet arrangement with small‐size electrode, (C) wall jet arrangement with large‐size electrode, (D) on‐capillary wire detection, (E) on‐capillary sputtered electrode detection. Side views are shown on the right‐hand side of the corresponding configuration.

In both off‐column and end‐column detection modes, precise alignment of the working electrode relative to the capillary outlet is critical. Accordingly, several spatial electrode configurations have been described, commonly referred to as in‐capillary, on‐capillary, and wall‐jet arrangements, which are schematically illustrated in Figure 2. The in‐capillary and on‐capillary configurations are almost ultimately restricted to off‐column detection, as positioning the electrode inside or directly on the separation capillary without proper electrical decoupling leads to substantial electric interference. In contrast, the wall‐jet configuration is the most commonly employed arrangement in end‐column detection. All alignment possibilities are discussed in detail below.

#### 4.2.1 In‐Capillary Detection

In in‐capillary detection, a microelectrode with a diameter of only a few micrometers—most commonly a carbon fiber electrode [1] or a thin metal wire—is inserted directly into the separation capillary (Figure 2A). This configuration provides superior sensitivity and minimal band broadening, as postcolumn dead volume is eliminated and analytes are detected before they can diffuse into a bulk reservoir. Owing to the small electrode dimensions (typically 5–10 µm carbon fibers), the charging current is exceptionally low, resulting in reduced noise and high coulombic efficiency [54].

With respect to spatial stability, once the microelectrode is positioned inside the capillary using an XYZ micromanipulator, its position remains fixed over time [2]. However, because such microelectrodes generate extremely low currents—often in the low picoampere range—the detection electronics must employ ultra‐low input bias current amplifiers [64] and ensure rigorous electrical isolation from the high‐voltage separation circuit to maintain an acceptable S/N ratio. In addition, this configuration may be susceptible to electrode breakage and bubble formation or impurity trapping from the BGE; therefore, careful optimization of experimental conditions and thorough buffer filtration are required.

To circumvent the need for electrical decoupling, a narrow capillary (e.g., 2 µm id) may be locally etched at the outlet to increase its diameter and accommodate a larger fiber microelectrode (Figure 2A, right). Under these conditions, even end‐column detection can be used, as demonstrated by Sloss and Ewing [65]. Nevertheless, this represents an exceptional case, since wall‐jet configurations are generally preferred for end‐column electrochemical detection.

#### 4.2.2 Wall Jet Capillary Detection

The wall‐jet configuration is the most widely adopted arrangement for end‐column detection (Figure 2B,C). Its primary advantages lie in its mechanical robustness and ease of alignment, especially with large electrode sizes, such as originally reported by Ye and Baldwin [66] (Figure 2C).

Larger electrodes tend to be less sensitive to small positioning variations, as their extended surface can intercept a greater fraction of the analyte zone, which may result in slightly higher collected currents [44]. However, this benefit is typically accompanied by a larger effective detection volume and increased analyte dispersion, leading to somewhat reduced separation efficiency compared with electrode dimensions closely matching the capillary inner diameter. Despite these theoretical concerns, practical studies by Matysik [44, 53] comparing electrodes of 25 and 500 µm diameter concluded that their analytical performance remained remarkably similar, suggesting that the wall‐jet's convective properties help mitigate the dispersion typically associated with larger surface areas.

Positioning the working electrode as close as possible to the capillary outlet in a wall‐jet arrangement is generally preferred because it helps limit postcolumn band broadening arising from diffusion and hydrodynamic dispersion after analytes exit the separation capillary. As the distance between the outlet and the electrode increases, some loss of separation efficiency and peak distortion may occur [67]. In the wall‐jet configuration, the perpendicular impingement of the solution from the capillary onto the electrode surface creates a high‐convection environment, which can enhance mass transport and provide a stable electrochemical signal [5]. Ultimately, the wall‐jet configuration is preferred for applications where extreme sensitivity is less critical than achieving highly robust and reproducible analytical behavior.

#### 4.2.3 On‐Capillary Detection

On‐capillary detection in CE–AD offers several advantages, most notably rugged operation, no alignment issues (for instance, for three separately created electrodes, the relative standard deviation was found to be 5% [68]), and high separation efficiency due to minimal postcolumn band broadening. However, on‐capillary detection presents several technical challenges. Integrating electrodes on the capillary requires complex fabrication and limits the ability to clean or replace the electrode, which may affect long‐term performance [2]. The proximity to the high‐voltage separation field can cause electrical interference, necessitating careful decoupling strategies. Despite these limitations, on‐capillary detection remains attractive for applications requiring high efficiency and miniaturized formats.

One example of on‐capillary detection is positioning of an Au wire across the id of the separation capillary and affixing it to the outer wall by the epoxy glue (Figure 5A) [68]. This design ensures that the electrode is fixed in position with excellent precision, the only disadvantage being that when it fails, one needs to replace the whole capillary, including the electrode. This, however, applies also for other on‐column detection approaches, for instance for the electrode sputtered on the capillary tip (gold or platinum) that has been applied by Voegel et al. [69, 70] (Figure 2E).

## 5 Electrode Materials for Neurotransmitter Analysis in CE–AD

**FIGURE 3:**
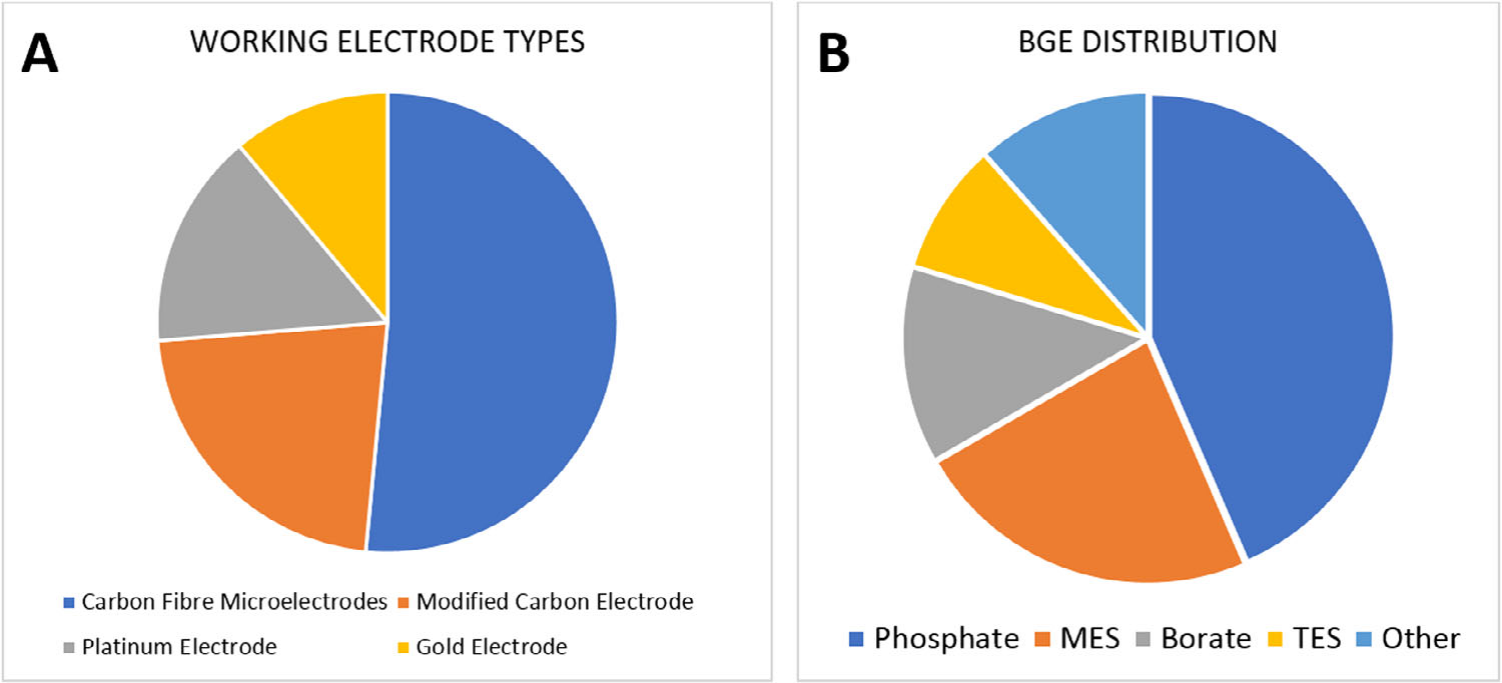
(A) Relative percentage use of various electrode materials across the CE–AD in neurotransmitter analysis. (B) Distribution of background electrolytes (BGEs) in neurotransmitter analysis by CE–AD. The data in this figure are based on a literature survey conducted in the Web of Science database in October 2025, comprising a total of 77 articles relevant to capillary electrophoresis with amperometric detection of NTs.

In AD, the choice of electrode material plays a crucial role in achieving sensitive, selective, and reproducible detection. Carbon‐based electrodes are the most widely employed due to their broad potential window, low background current, and chemical stability [71]. They are particularly suited for detecting catecholamines and indoleamines that undergo oxidation at moderate potentials. Various forms of carbon—such as carbon fiber [1, 51], glassy carbon [72], or carbon paste [73] have been successfully applied, often further modified to enhance electron‐transfer kinetics and minimize fouling by biological matrices. Surface modifications with carbon nanotubes [74, 75], metal nanoparticles, or conductive polymers [76] significantly improve analytical performance by increasing active surface area and lowering oxidation overpotentials. Gold (Au) and platinum (Pt) electrodes are widely used in fundamental electrochemical studies due to their well‐defined crystalline surfaces and reproducible surface chemistry. However, their accessible potential window in aqueous media is generally narrower than that of carbon‐based electrodes [77], particularly boron‐doped diamond (BDD) [78], which are more suitable for analytes requiring high oxidation potentials. Nevertheless, Au and Pt electrodes have been applied in numerous articles on NT analysis. Figure 3A shows an overview of the percentage of the electrode material used in all reviewed articles. Clearly, the carbon‐based materials form more than 70% of all detection electrodes, while Pt electrodes have a slight edge over the Au electrodes. In the following sections, the principal electrode materials applied in neurotransmitter analysis are examined in detail, with particular emphasis on their electrochemical characteristics and a quantitative comparison of reported analytical figures of merit, including limits of detection, calibration range, selectivity, and operational stability.

### 5.1 Carbon Fiber Electrodes

Carbon fiber electrodes (CFE) are among the most widely used working electrodes in CE–AD due to their simple fabrication, favorable electrochemical properties, and excellent compatibility with narrow‐bore capillaries. Carbon fibers (typically 5–10 µm diameter) are manufactured from polyacrylonitrile precursors by stabilization in air followed by high‐temperature carbonization under inert atmosphere [71, 79]. Their major advantages include low background currents, wide anodic potential windows, good sensitivity toward catecholamines and indoleamines, and favorable signal‐to‐noise ratios [71]. However, they are mechanically fragile and have a limited long‐term reproducibility compared with other carbon‐based materials because their surface cannot be renewed by simple polishing, as is the case with carbon microdisk or metal electrodes [80].

In Wallingford and Ewing's [1] original article, which used a 10 µm CFE with a porous‐glass decoupler, the authors showed an off‐column detection with high separation efficiency (180 000 plates for catechol/catecholamines) in a 75 µm id capillary and pmol sensitivity for NTs using 3‐(cyclohexylamino)‐1‐propanesulfonic acid BGE at pH 10. Moving to smaller dimensions, they then showed amperometric detection on 26 µm id capillaries with sub‐femtomole mass detection for several catechols [51]. Micellar electrokinetic chromatography (MEKC) was used to add selectivity, establishing that CFE pairs naturally with both capillary zone electrophoresis (CZE) and MEKC, albeit with MEKC trading some electrochemical sensitivity for separation selectivity depending on SDS concentration [81]. Eventually, they extended CE–AD to even smaller, 12.7 µm id capillaries, enabling attomole‐level detection and single‐cell neurotransmitter analysis [82] and finally to 9 µm id capillaries, where end‐column amperometry (still using CFE) achieved 0.7 amol for serotonin and low‐amol limits for other NTs [83].

Although all of the aforementioned studies employed off‐column detection with electrical decoupling, reducing the capillary inner diameter leads to a corresponding decrease in electrophoretic current, thereby minimizing or even eliminating the need for a decoupler. Recognizing this, Sloss and Ewing [65] introduced a truly decoupler‐free end‐column CE–AD configuration in 2 µm capillaries. They further optimized the system by etching the capillary outlet to self‐align the carbon fiber at the bore, raising coulometric efficiency to approximately 91% with best‐case LODs of 10–11 amol approaching the earlier off‐column sensitivity without the use of fragile decouplers.

The series of articles by Woods and Ewing represents the next evolutionary stage by pushing CE–AD in end‐column detector configuration into sub‐micrometer id capillaries (770 and 430 nm) and solving the detector limitations that arose at this extreme scale. In the first article [45], peak efficiencies dropped dramatically in nanocapillaries (500–5000 plates) due to detector dead volume and wall interactions. The subsequent paper [46] directly addressed the efficiency problem by redesigning the detector by etching both the capillary outlet and flame‐etching the carbon fiber electrode conical tip. Generally, the sensitivity improved from attomole detection in 9 µm id capillaries [83] to zeptomole levels [46] and eventually allowed detection of dopamine in intact PC12 mammalian cells [84]. These studies map a continuous six‐to‐nine‐order‐of‐magnitude improvement in mass detection sensitivity for catecholamines, culminating in quantitative cytoplasmic neurotransmitter analysis at the single‐cell level.

While simple etching, the end of an extremely narrow bore separation capillary allows CE–AD without electrical decoupling and affords extremely low mass detection limits, such capillaries are not suitable for real sample injection due to the possibility of easy capillary clogging. To address this limitation, Lunte's group [56] introduced an end‐column “decoupler” by casting a short, large‐bore Nafion tube at the outlet of a 50 µm id separation capillary with the CFE positioned inside the Nafion sleeve. Although the achieved LODs were on the order of 3 nM for catecholamines, this approach prioritized robustness and compatibility with complex biological matrices over maximum sensitivity, demonstrating the practical advantages of using larger id capillaries. Around the same time, Chen and Whang [55] introduced a porous CA joint as an alternative decoupling material, formed by coating a fractured capillary with a thin CA film that allowed ionic conduction but excluded larger analytes. This joint combined electrical isolation with essentially zero dead volume, producing over 90 000 theoretical plates and LODs as low as 6–14 fmol for catecholamines, demonstrating a low‐cost, durable, and easily fabricated off‐column interface. Other pragmatic decoupling innovations include the “etched‐joint” (HF‐thinned, porous wall segment made directly on the capillary), which isolates the detector without adding a separate sleeve and works on 50 µm id capillaries for catechols/diphenols and is simpler to build than porous glass or wrapped polymers while retaining the off‐column topology [85]. Later, Qian et al. [86] demonstrated an integrated HF‐etched end‐column decoupler in a precisely aligned CFE detection cell that operated at 600 mV versus Ag/AgCl with only 50 fA noise even under 10–20 µA separation current. This allowed attomole mass and nanomolar concentration LODs for DA in 1‐min brain microdialysates—an important step toward in vivo temporal resolution.

In biological applications, the same CFE platforms enabled scanning electrochemical detection through elution, confirming peak identity voltammetrically and quantifying femtomole amounts of DA from single neurons (344–461 fmol per cell), underscoring the high signal‐to‐noise at truly tiny sample loads [87]. Ferris et al. [88] also used scanning electrochemical detection, where the working potential was cyclically varied during the electrophoretic run to obtain full voltammetric information for each migrating zone. This innovation improved chemical identification of overlapping catechol peaks and achieved detection limits of 360–740 amol for catechols on 5 µm carbon fibers. Finally, Weng et al. [89] demonstrated that pairing end‑column CFE detection with field‑amplified sample stacking (FASS) yields sub‑nanomolar LODs for monoamines in urine (e.g., 0.04–0.16 nM in standards; practical 50‑fold gain in real urine after dilution), bridging single‑cell‑scale sensitivity and clinical matrices.

To conclude, CFEs combine favorable electrochemical properties with excellent geometric compatibility across a wide range of capillary dimensions, from tens of micrometers down to the sub‐micrometer scale. This evolution has resulted in substantial gains in sensitivity, directly linked to the progressive reduction in capillary inner diameter. Further miniaturization into nanocapillaries required redesign of the detector geometry to mitigate efficiency losses, but ultimately pushed detection limits into the zeptomole range and enabled direct measurements in intact cells, representing several orders‐of‐magnitude improvement in mass sensitivity. However, such extreme miniaturization compromises robustness and sample compatibility, driving the development of practical decoupling strategies (e.g., Nafion sleeves, CA joints, etched interfaces) that trade some sensitivity for reliable analysis of complex biological matrices.

### 5.2 Carbon Disk Electrodes

Bulk carbon electrodes, including glassy carbon and graphite, represent a robust and chemically inert type of working electrode. Glassy carbon is produced by high‐temperature pyrolysis of polymeric precursors (phenolic resins), resulting in a dense, nongraphitizing carbon structure with low porosity and good electrical conductivity [71]. When precise alignment with the capillary outlet is less critical, these electrodes are used because, compared with carbon fiber electrodes, they offer improved mechanical stability, longer operational lifetimes, and easier surface renewal through polishing [80]. On the other hand, their larger surface area typically leads to higher background currents and increased detection volumes, which may reduce separation efficiency and signal‐to‐noise ratios relative to microelectrodes.

In most of the published articles that use carbon disk electrodes, end‐column AD in narrow bore capillaries with id between 11 and 25 µm was applied, with a few exceptions published by Matysik's group. In their first article, Matysik et al. [90] demonstrated that end‐column amperometric detection using carbon microdisks (0.4 mm) could be performed even in large bore capillaries. The key innovation was the demonstration that stable end‑column detection can be achieved in conventional 50 µm capillaries without decoupling, contrary to earlier assumptions, provided a low conductivity BGE is utilized. The system achieved LODs of 50 nM for 5‐HT. In these two seminal papers [44, 90], 10 mM phosphate BGE was used, while nonaqueous BGEs were used for 75 µm id capillaries [53]. A major contribution of the series of works by Matysik et al. is the fact that this simplified geometry preserved separation efficiency while maintaining practical robustness. The results challenge the assumption that decouplers are mandatory for capillaries above 25 µm id, thereby expanding CE–ED applicability to routine capillary formats. Worth noting in this context is the additional simplification of the AD design presented by Kappes and Hauser [91], who converted the three‐electrode cell to a two‐electrode design by using the electrophoretic ground as a polarized pseudo‐reference and a graphite electrode, although in 25 µm id capillaries.

The majority of other authors, however, used capillaries with id below 25 µm, which provided better flexibility in using higher‐concentration BGEs. In these studies, phosphate buffers in the range of 50–200 mM were commonly utilized. Most of the following reports also employed a 300 µm carbon disk electrode in wall‐jet geometry. For example, the group of Jiannong Ye [92] used a 300 µm carbon disk in wall‐jet geometry to detect MNTs (DA, NE, E, and 5‐HT) and their metabolites in a system coupled with microdialysis sampling from the rat caudate nucleus. A relatively high phosphate BGE concentration (160 mM, pH 6.5–7.0) was used with moderate sensitivity in the low micromolar range for most of the analytes. Later, the same group [93] employed a similar 300 µm electrode disk but introduced a micro‐injector to handle sub‐µL pineal gland extracts, analyzing NTs (MT, 5‐HT, 5‐HTrp, Trp) in 200 mM phosphate BGE (pH 8.0) with LODs of 0.03–0.13 µM. Wang et al. [94] used a comparable carbon disk electrode with a 25 µm id capillary and phosphate at pH 7.0 for the simultaneous determination of LDME, L‐DOPA, and DA in rat serum. The achieved limits of detection were comparable to those reported by Jiannong Ye's group [92, 93], demonstrating that carbon disk electrodes remain effective even in complex biological matrices.

Because the concentration sensitivity using the previous systems was relatively low, Wu et al. [95] advanced the approach by integrating MEKC with end‐column AD at a 300 µm carbon disk and electrokinetic stacking, reaching low, 9.7–41.8 nM LODs. This was a significant improvement, additionally confirming the wall‐jet carbon disk's suitability for MEKC and for simultaneous quantification of precursors and metabolites without derivatization. Tang et al. [96] retained the same end‐column carbon disk format (300 µm) but paired it with a dynamic pH‐junction preconcentration (acidic sample plug into 150 mM boric acid BGE, pH 10.33), achieving 100‐fold sensitivity gain and LODs of 5.34–68.3 nM for DA, NE, E, 5‐HT, tyramine (TA), and tryptamine (TrA)—showing that stacking, not the electrode per se, was the dominant lever for nanomolar detection with a bare carbon disk. Fang et al. [97] replaced amperometry with fast‐scan cyclic voltammetric (FSCV) detection after CE separation of a single Drosophila larva brain, using a similar carbon microelectrode and field‐amplified sample stacking, aiming at even lower LODs. The method reached 1–4 nM LODs for DA, 5‐HT, TA, and octopamine (OA) while providing compound‐specific cyclic‐voltammogram fingerprints.

In general, the application of carbon disk electrodes in CE–AD has been dominated by end‐column configurations, typically in narrow‐bore capillaries (<25 µm id), although the work of Matysik et al. demonstrated stable operation even in 50–75 µm capillaries without electrical decoupling when low‐conductivity BGEs and appropriate potential compensation were applied. Subsequent studies confirmed the compatibility of carbon disk electrodes with complex biological matrices and high‐ionic‐strength phosphate buffers, while innovations such as wall‐jet geometry, simplified two‐electrode setups, and integration with microdialysis sampling enhanced practical usability. Ultimately, major gains in sensitivity—from micromolar to low‐nanomolar levels—were achieved through stacking techniques, MEKC coupling, and advanced detection modes such as FSCV, underscoring that preconcentration strategies are the dominant factor governing detection limits in these systems.

### 5.3 Composite/Chemically Modified Carbon Electrodes

**FIGURE 4:**
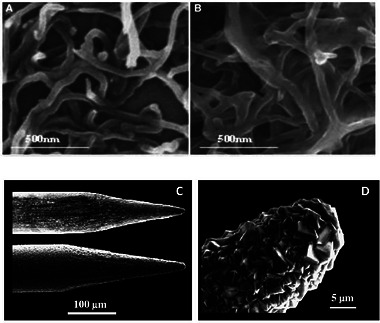
SEM images of (A) MWCNTs, (B) MWCNTs@Pdop, (C) an electrochemically sharpened Pt wire (top) and Pt wire covered with a polycrystalline BDD film (bottom), (D) the tip of a 76 µm Pt wire covered with a BDD film. *Source*: Reproduced from *Electrophoresis* [74] with permission from Wiley and from *Diam Relat Mater* [102] with permission from Elsevier.

Chemically modified carbon electrodes (CMCEs) extend the capabilities of conventional carbon materials by introducing functional layers, coatings, or immobilized mediators onto the electrode surface. Modifications include, for instance, polymer films [76, 98], nanoparticles/nanotubes [74], enzymes, or covalently attached functional groups, typically applied to carbon fibers or glassy carbon substrates. One example of such modification is shown in Figure 4A,B, where multiwalled carbon nanotubes (MWCNTs) were coated on a carbon disk electrode [74]. The key advantage of CMCEs lies in tunable surface chemistry, which allows improved selectivity, reduced fouling, and catalytic signal amplification. These benefits are offset by increased fabrication complexity, potential instability or leaching of the modifying layer, and reduced reproducibility compared with unmodified carbon electrodes.

Early work with polymer‐modified films demonstrated the advantages of chemical modification of the bare carbon electrodes. Chen et al. [98] used a polyhistidine film to increase the amperometric peak currents of catechol NTs and shift hydrodynamic voltammograms to lower potentials. CE separations of DA, E, and catechol yielded LODs of 6 nM for DA and 8 nM for E. Compared with bare carbon, the polymer increased currents fivefold at the same potential and allowed operation at 0.2–0.3 V, which helped to suppress background and electrode passivation in continuous runs.

Sol–gel carbon composite electrode (CCE), where graphite is dispersed in a silica network to produce low‐capacitance, low‐noise detectors, was used in either wall‐jet/end‐column cells or as integrated on‐capillary tubular electrodes. They are fabricated by dispersing graphite powder in a methyltrimethoxysilane‐based sol–gel matrix and packing it into a glass capillary. The sensitivity of sol‐gel CCE was, however, only moderate compared with the previous work by Chen [98] and other electrode designs. Hua and Tan [73, 99] showed that a simple anodic pretreatment of the sol–gel CCE reproducibly boosts catecholamine currents (12–18× in CV) and supports linear ranges of 1–400 µM for DA, NE, E, and catechol, with amount LODs of 220–350 amol but concentration LODs around 0.7 µM (DA) and 1.2 µM (catechol). The original wall‐jet arrangement was later implemented as an integrated tubular detector with no need for decoupling. This on‐capillary integration eliminated micropositioners, improved day‐to‐day reproducibility (RSD 2.3%–3.5% for 15 injections), and provided month‐scale usability before capillary replacement. The highest sensitivity with sol–gel CCE was achieved by Sun et al. [100] who reported E LODs of 30 nM with wide linearity and 20% of the noise seen at the carbon paste electrode, bringing performance close to the 33 µm CFEs but with simpler construction and lower drift. From a decoupling standpoint, both the wall‐jet CCE and the integrated tubular CCE operate as end‐column schemes with the outlet held at ground; neither requires a decoupler, which reduces dead volume and setup complexity relative to classical end‐column cells.

Another approach to prepare a paste‐type electrode was applied by Chicharro et al. [75]. A carbon‐nanotube paste electrode (CNTPE) incorporated into a purpose‐built end‐column cell enabled detection of DA, NE, E, DOPAC, and ascorbic acid (AA) at just +0.40 V with enhanced currents versus graphite paste, but low‐µM range LODs (e.g., DA 0.7–1.8 µM). The detector design intentionally avoided micropositioners by using a 0.5 mm working electrode and a 100 µm capillary‐to‐electrode gap. The same group also electrogenerated a melanin‐type (L‐DOPA‐derived) polymer on graphite/glassy‐carbon disks to create an anion‐rejecting, cation‐permissive film [76]. This allowed deliberate addition of millimolar AA to the detection cell to amplify catecholamine currents without the usual AA interference, while operating at +0.70 V for DA/NE/E. This approach demonstrated selective oxidation of cationic catecholamines while rejecting anionic interferents such as AA and DOPAC; however, at the cost of sensitivity (DA LOD was threefold higher than at the bare carbon disk). Finally, in an excellent contribution by Liu et al. [74], multiwalled carbon nanotubes (MWCNTs) were functionalized with polydopamine (Pdop), providing abundant catechol/amine groups for metal anchoring. Platinum (Pt) nanoparticles were then electrodeposited onto MWCNTs@Pdop to form Pt/MWCNTs@Pdop (see Figure 4A,B). Such combinations have pushed concentration LODs below 1 nM, which are so far the lowest achieved with CMCEs. For 5‐HT, DA, NE, and E, the LODs were between 0.3 and 0.92 nM. Importantly, the study moves beyond model solutions and applies CE–AD to real neurochemical monitoring in brain tissue, illustrating translational potential for studying stress‐induced neurochemical alterations by quantifying monoamines in rat hippocampus and hypothalamus before and after noise stimulation.

Chemically modified carbon electrodes in CE–AD offer some advantages over bare carbon electrodes, such as lower operating potentials with higher catalytic currents, tunable selectivity that may suppress the interferents, but these benefits come with trade‐offs such as higher LODs compared with bare carbon in clean samples, potential film degradation over long use, and the need to optimize coating parameters to balance permeability and selectivity. In concentration‐LOD terms, the best LODs were achieved at Pt/MWCNTs@Pdop, where four monoamines were detected at 0.3–0.92 nM. Together, these results map a clear evolution: from polymer films that enabled lower‐potential CE–AD but only micromolar LODs, through robust sol–gel/tubular architectures that simplified decoupling toward CNT/metal‐NP hybrids that deliver sub‐nanomolar LODs in standard end‐column cells and could be used, for instance, in real samples from rat hippocampus and hypothalamus.

### 5.4 Boron Doped Diamond Electrodes

Boron‐doped diamond (BDD) electrodes are fabricated by incorporating boron atoms into diamond films grown by chemical vapor deposition (CVD), yielding a highly conductive yet chemically inert electrode material [101]. The deposited films consist of polycrystalline diamond with grain sizes depending on growth conditions and can be formed as thin films on conductive substrates, for instance, platinum [102]. Their most significant advantages include an exceptionally wide potential window, extremely low background currents, high resistance to fouling, and outstanding chemical and mechanical stability [78]. These properties make BDD electrodes particularly attractive for complex biological matrices and high‐potential oxidations. However, their disadvantages include higher cost, more complex fabrication [102], and generally lower intrinsic sensitivity toward some neurotransmitters compared with carbon‐based electrodes, which may require application of higher detection potentials [103].

In the first report on BDD implementation in CE–AD of NTs, Shin et al. [104] used BDD microline electrodes as working electrodes for end‐column amperometric detection. The authors achieved high plate counts (155 000 for DA) and sub‐25 nM concentration LODs for DA/NE/E under mild pH (30 mM MES, pH 5.7). The BDD electrodes were compared with a carbon fiber disk electrode, and despite the larger area, they exhibited significantly lower background noise (0.5–1 pA). Importantly, no mechanical polishing was required between runs due to its strong resistance to fouling and low adsorption of organic analytes. Cvačka et al. [102] independently fabricated conical BDD microelectrodes by CVD on sharpened Pt wires (see Figure 4C,D) and, with a standard end‐column cell (75 µm id capillary; pH 6 phosphate), reported linear ranges spanning four orders of magnitude and only slightly higher LODs of 78–150 nM for dopamine/catechol. Again, the peak potentials for catechols and catecholamines were highly reproducible across electrodes. No pretreatment was required, and no electrode fouling or deactivation was observed during extended use. This makes the BDD electrodes very suitable for biological samples, as demonstrated in later publications.

Thus, moving from standards to biological samples, Park et al. [103] used the same conical BDD microelectrode and achieved 40–250 nM LODs for NTs and metabolites without any conventional pretreatment, underscoring BDD's fouling resistance relative to carbon fiber. For endogenous analytes in tissue, Novotný et al. [105] and Quaiserová‐Mocko et al. [106] validated an end‐column, decoupler‐free method using a BDD microelectrode together with borate complexation (250 mM borate, pH 8.8) and off‐line SPE cleanup.

In conclusion, the performance of BDD compares favorably with typical glassy‐carbon end‐column detectors, particularly when the task involves repeated injections or complex matrices that readily foul conventional carbon‐based electrodes. In particular, BDD surfaces require no mechanical or electrochemical pretreatment, which is advantageous for robust operation and maintaining a stable response during repeated injections of biological samples, such as tissue homogenates, enabling reliable long‐term operation.

### 5.5 Platinum Electrodes

Pt electrodes are noble‐metal working electrodes valued for their chemical robustness, high conductivity, and catalytic activity [107]. Pt is typically used as wire electrodes or microfabricated thin films in end‐column or wall‐jet detection configurations. The Pt electrodes offer excellent stability and reproducibility—for example, a 50 µm Pt disk electrode in an end‐column CE detector achieved amol‐level detection limits for catecholamines with only 2%–5% relative standard deviation in peak currents [67]. However, relative to carbon and diamond electrodes (i.e., BDD), Pt exhibits a narrower usable potential window in aqueous media and higher background currents due to surface oxide formation [108]. Additionally, Pt surfaces are prone to fouling and catalytic side reactions [40], which can complicate quantitative analysis of neurotransmitters and reduce long‐term stability in complex biological samples.

Early work by Chen and Huang [67] introduced a compact end‐column cell employing a 50 µm Pt disk electrode in a wall‐jet configuration and a 5 µm id separation capillary. The electrode was a wire enclosed in a short fused‐silica capillary segment and embedded in PTFE tubing, eliminating the need for a micromanipulator and microscope. This arrangement minimized dead volume and mechanical drift, providing highly reproducible detection with remarkable sensitivity with LODs as low as 3.0 amol for DA and 5.2 amol for catechol using a 20 mM MES BGE (pH 6.0).

Matysik and Backofen [90] demonstrated that end‐column amperometric detection could be reliably performed in 10‐fold larger id capillaries (50 µm) without any decoupler using a Pt microdisk working electrode positioned directly at the outlet. Using a low‐conductivity 10 mM phosphate BGE (pH 8.0), they successfully separated 5‐HT and other analytes with high efficiencies and validated stable Pt operation under high‐voltage conditions without decoupling. Voegel and Baldwin [69] subsequently advanced the concept of integrated detection by sputtering thin Pt (or Au, see the following section) films directly onto fused‐silica capillaries, creating the first on‐capillary electrodes (OCEs). These OCEs eliminated mechanical alignment requirements and could also operate decoupler‐free. Their follow‐up study [109] extended the concept to dual‐parallel on‐capillary electrodes, fabricated by sputter‐coating two identical capillaries with Pt. This configuration enabled simultaneous AD at two independent potentials or with different electrode materials. The approach also allowed differential detection of DA and NE and enzymatic sensing of glucose when coupled with glucose oxidase.

**FIGURE 5:**
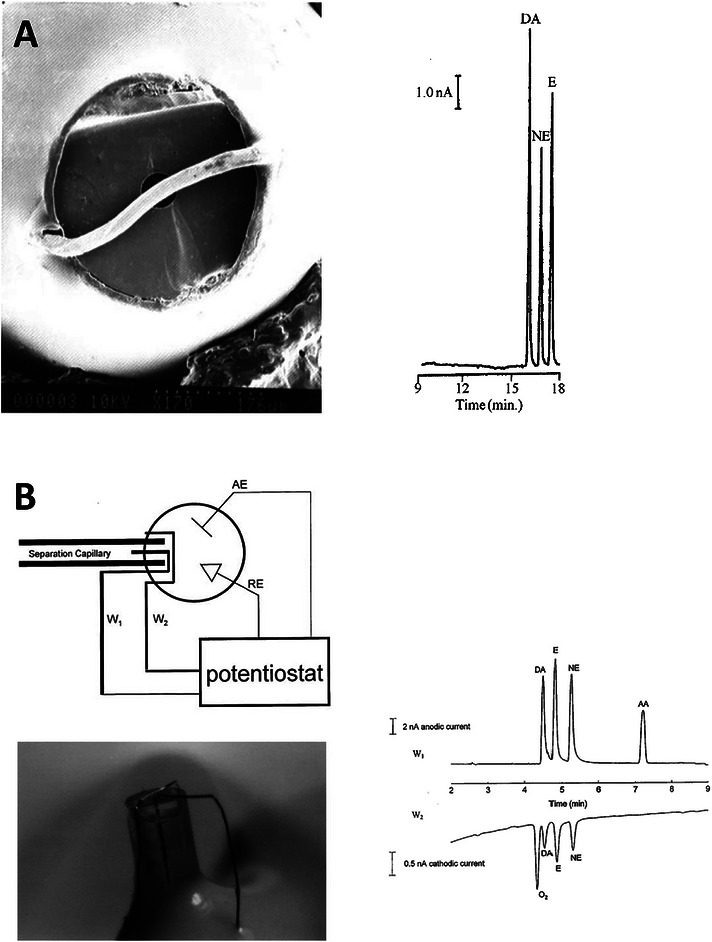
(A) Scanning electron micrograph of the Au wire on‐capillary microelectrode positioned directly across the capillary outlet and the corresponding separation of NTs. (B) Schematic and photograph of an integrated on‐capillary dual Pt electrode design with corresponding electropherogram showing separation of NTs on first (W1, *E* = + 0.950 V) and second (W2, *E* = −0.300 V) electrodes. *Source*: (A) Reproduced from *Analytical Chemistry* [68] with permission from American Chemical Society. (B) Reproduced from *Electroanalysis* [110] with permission from Wiley.

Holland et al. [110] further refined this concept by constructing an integrated dual Pt wire‐wire electrode fixed directly to the capillary tip in a true series configuration (see Figure 5B). The upstream (generator) electrode was maintained at +0.95 V and the downstream (detector) at –0.30 V, enabling direct evaluation of redox reversibility for phenolic and catecholamine compounds. The integrated dual‐wire design provided high collection efficiencies (0.35–0.40 for reversible redox species) and eliminated the need for postrun alignment. A further simplification of alignment was achieved by Jin et al. [111], who developed an end‐column detection system in which a 25 µm Pt wire was inserted directly into the capillary outlet and stabilized by a liquid junction that simultaneously acted as the electrical ground. The detector provided low‐nanomolar sensitivity for catecholamines and excellent reproducibility (RSD < 2.5%), bridging traditional wall‐jet and later integrated electrode systems. Wallenborg et al. [112] systematically evaluated the effects of separation voltage and electrode spacing on Pt‐based end‐column detection, demonstrating that potential shifts of up to +130 mV occurred with increasing field strength (15–30 kV) and could be minimized through fracture‐type decoupling. Osbourn and Lunte [57] then introduced a laser‐etched, CA‐coated, on‐column decoupler combined with a 25 µm Pt wire electrode. This design dissipated separation current efficiently, avoided cation‐exchange interference, and achieved sub‐nanomolar limits of detection (0.2 nM for DA) with four‐order linearity (1 nM–10 µM).

Across these studies, Pt electrodes were used from end‐column disk electrodes to permanently fixed on‐capillary and dual‐wire configurations, and ultimately to systems combined with high‐performance cellulose acetate decouplers. Dual Pt electrode configurations enabled collection‐efficiency measurements and selective detection of reversible species, expanding CE–EC from simple amperometry toward more detailed electroanalysis. Although Pt typically provides only µM‐level LODs in nondecoupled formats, its electrochemical stability, polishability, and compatibility with high‐current separations enabled reproducible operation under conditions where carbon fibers posed limitations.

### 5.6 Gold Electrodes

Au electrodes are also commonly used in CE–AD due to their excellent electrical conductivity, well‐defined surface chemistry, and ease of microfabrication. Au working electrodes can be fabricated as wires, thin films, or microelectrodes and are compatible with end‐column, wall‐jet, and microchip CE–AD configurations. Their major advantage lies in their ability to support self‐assembled monolayers and other surface modifications, enabling controlled surface chemistry and selective detection strategies [113]. Au electrodes also exhibit fast electron‐transfer kinetics for certain analytes [114]. Again, compared with carbon‐based materials and similarly to Pt, Au has a narrower anodic potential window [115], higher susceptibility to surface fouling by adsorption of organic species [116], and generally higher background currents, which can limit its ultimate sensitive detection of NTs [108, 115].

In one of the earliest integrated on‐capillary electrode designs, Zhong and Lunte [68] fixed a 25 µm Au wire directly across the capillary outlet (see Figure 5A), eliminating micromanipulator alignment and allowing routine neurotransmitter measurements. With 50 µm id capillaries and acidic citrate BGE (20 mM, pH 2.5), they reported 0.12 µM LOD for DA in on‐capillary mode (S/N = 2), versus 0.50 µM with an in‐capillary Au electrode. The improvement of the sensitivity was assigned to reduced noise due to better electrical grounding compared with in‐capillary insertion. Similarly to Pt OCEs, Voegel et al. [109] sputtered thin Au (0.3 µm) films directly onto the capillary tip in an arrangement that required no alignment during operation. A dual‐parallel electrochemical detection system based on two identical fused‐silica capillaries was used, and moderate LODs in dual mode were 0.76–1.35 µM, only slightly higher than single‐capillary operation (0.42–0.91 µM). Perhaps the most striking sensitivity gain with Au came from geometry‐driven redox‐cycling by Chen et al. [117] who combined an on‐capillary Au film electrode with a facing disk electrode to create a thin‐layer, parallel‐opposed generator–collector cell at the capillary outlet. For electrochemically reversible DA in pH 6.9 phosphate and a 12.5 µm id capillary, the configuration yielded a 12 nM LOD (4.2 amol), demonstrating that clever cell design can fully offset Au's intrinsic sensitivity disadvantages. It should be noted, though, that even if this LOD is lower, it is still not reaching the LODs achieved with other types of electrodes (carbon fiber, BDD).

To simplify the capillary to electrode alignment, Cheng et al. [118] adapted a fiber‐optic MT connector as a plug‐and‐play holder. The connector allowed rapid and reproducible assembly time (<10 s) and facilitated routine polishing and reassembly of the working electrode. While the detection limits were in the sub‐micromolar range, the principal advancement lies in mechanical robustness, reproducibility after repeated polishing cycles, and controlled micrometer‐scale spacing, addressing one of the major practical limitations of conventional end‐column CE–EC systems. In the same vein, regarding electrode to capillary alignment, Goto et al. [119] designed a handy low‐cost end‐column cell using a mixing joint that stabilized capillary–electrode geometry without micropositioners. The advantage is again in rapid assembly, adjustable spacing, and easy electrode exchange. The system proved suitable for routine multiclass analyte determination, while the Au electrode provided good sensitivity for catecholamines (23–44 nM (11–21 amol)) for DA, E, and NE. Eventually, Klett et al. [120] introduced a “deviceless” decoupling scheme that placed Au microband working and quasi‐reference electrodes only 10 µm apart on a microfabricated detector chip, so both electrodes experienced essentially the same separation field perturbation. They could hold the detection potential just negative of Au‐oxide formation (−0.6 V), thus avoiding rapid fouling during DA oxidation; however, paying the price of low sensitivity (LOD of 13 µM for DA). Still, the approach elegantly solved potential drift and alignment stability in a format that is highly amenable to microreplication. Finally, a much more recent study by Feito et al. [121] integrated an automated sequential injection analysis—CE manifold with amperometry. They isolated the fluidic manifold from HV with an air plug and reached 0.3 µM LODs for DA and E. The system was used for online monitoring applications, achieving sub‐µM LODs while maintaining excellent reproducibility and allowing long‐term unattended operation (825 continuous runs [>1 week]).

Across the studies employing Au electrodes, the configurations evolved from early on‐capillary designs [109, 117] toward simplified and more robust arrangements utilizing commercial optical or mixing connectors [118, 119], enabling rapid assembly, adjustable electrode positioning, and straightforward electrode replacement. Although the achieved sensitivity was generally moderate compared with carbon‐based systems, these developments emphasize the practical advantages of Au electrodes in terms of mechanical stability, reproducibility, and ease of implementation. Importantly, their unique ability to support self‐assembled monolayers and other surface modifications enables precise control over interfacial chemistry and facilitates selective detection strategies. This feature is particularly promising for tailoring electrode surfaces toward specific analyte classes and mitigating fouling effects in complex biological matrices.

### 5.7 Multielectrode Designs

Multielectrode designs in CE–AD have significantly improved sensitivity and robustness, primarily through redox cycling. This signal‐amplification strategy employs a dual‐electrode system (generator and collector) placed in proximity (typically within a few micrometers) to repeatedly “recycle” an analyte molecule between its oxidized and reduced forms. Rather than undergoing a single electron‐transfer event before leaving the detection zone, the analyte shuttles between the electrodes multiple times, resulting in substantial signal enhancement [122, 123]. These configurations have been fabricated using a variety of electrode materials, including carbon, Pt, and Au.

Carbon‐film interdigitated ring arrays (IDRAs) placed in a thin‐layer, radial wall‐jet flow cell allow CE effluent to sweep radially across concentric generator–collector microbands. Using this approach, Liu et al. [124] obtained high collection efficiencies (ca. 65% for catechol) and measurable redox cycling, translating into sub 100 nM detection limits for NTs—most notably 15 nM (DA), 23 nM (E), and 38 nM (NE) in dual (generator–collector) mode. Building on the same microfabrication logic but targeting still smaller volumes, the same group [125] miniaturized a sub‐nanoliter wall‐jet detector that accommodates an interdigitated microarray in front of the capillary outlet and demonstrated clear redox‐cycling enhancement for DA, evidencing that the applied geometry supports both strong signal amplification and very small effective volumes.

Pt interdigitated electrode (IDE) arrays have also been used as true multielectrodes that redox‐cycle the NTs in situ while enabling chiral CE using cyclodextrins. By poising seven IDE fingers oxidatively and one reductively, Male and Luong [126] observed clear amplification at the “collector” and could detect and assign six enantiomers of NE, E, and isoproterenol in a single run with LODs in the micromolar range. Finally, a hybrid parallel‐opposed dual‐electrode design that combines an on‐capillary electrode with a downstream disk (so the analyte shuttles between them) presented by Chen et al. [127] achieved subnanomolar quantitation of DA, E, and NE (LODs of 0.41 nM (DA), 0.14 nM (E), and 0.16 nM (NE)) directly in 5×‐diluted urine without cleanup or preconcentration. This was achieved by leveraging redox recycling for both signal gain and matrix discrimination, with isoproterenol as an internal standard.

While the previous examples focused on signal amplification, achieving sub‐nanomolar LODs, multielectrode arrays can also be used for electrical isolation. In contrast to carbon‐based recycling arrays, Klett et al. [120] pursued Au microband arrays positioned end‐column and introduced a “deviceless” decoupling concept. In this configuration, the microband array serves a dual purpose: it acts as the electrochemical sensor while simultaneously functioning as the system's electrical ground. The first few electrodes in the array encounter the HV separation field first and act as a distributed ground. This dissipates the electric field before it can interfere with the final electrodes in the array used for the amperometric readout. While this simplifies the hardware interface, it often results in higher background noise compared with physical decouplers, which is why these “deviceless” schemes typically report micromolar rather than nanomolar LODs. This “deviceless” concept (rooted back to the bipolar electrode observations of Lu and Cassidy [128]) has been further expanded into “potentiostatless” microfluidic systems by Ordeig et al. [129], where the separation field itself is harnessed to drive the electrochemical reaction across an array of Au microbands.

Overall, multielectrode CE–AD configurations provide a highly effective route to enhance sensitivity and functionality, with generator–collector designs enabling substantial signal amplification through redox cycling and achieving sub‐nanomolar detection limits for neurotransmitters in complex matrices. These systems also offer inherent selectivity toward electrochemically reversible species, improving performance in real samples. In contrast, Au microband arrays operating in “deviceless” or potentiostatless modes emphasize simplicity and integrated decoupling, but typically at the cost of higher noise and micromolar LODs. Thus, a clear trade‐off exists between sensitivity and instrumental simplicity. Future progress will likely aim to combine efficient redox cycling with simplified architectures to achieve both high sensitivity and practical integration.

## 6 Background Electrolytes in CE–AD for Neurotransmitter Analysis

A brief overview of the major BGEs used in NT analysis by CE–AD is shown in Table 3. For a more detailed overview, Table S2 provides the list of all papers from this review with corresponding separation conditions, sorted by the used BGE. An analysis of all articles shows that researchers consistently favored only a few BGE systems (see Figure 3B), with phosphate‐based BGEs being used most often.

**TABLE 3:** The most common BGE used in CE–AD analysis of neurotransmitters.

BGE type	Typical pH range	Best LODs achieved	References
Phosphate	5.5–8.0	0.03–0.09 nM [74]	[51, 57, 69, 73, 74, 81, 85, 86, 89, 90, 92, 93, 94, 95, 96, 97, 98, 99, 100, 102, 109, 112, 117, 118, 119, 124, 125, 139, 140, 141, 142, 143, 144]
MES	5.6–7.0	0.14–0.4 nM [127]	[51, 55, 62, 65, 67, 82, 83, 84, 87, 88, 91, 104, 120, 127, 134, 145]
Borate	8.8–10.3	25–32 nM [96]	[42, 72, 75, 76, 96, 103, 106, 110, 135]
TES	7.0–7.4	400 zmol [45, 46]	[45, 46, 136, 142, 146, 147]
Other	2.5–10.0	3 nM [56]	[1, 56, 68, 121, 126, 137, 138]

Phosphate BGEs (typically 10–200 mM, pH 6–7.4) are popular because they mimic physiological pH, provide strong buffering capacity, and are compatible with biological samples, microdialysis fluids, and enzyme‐based detection [130]. Their electrochemically inert nature allows integration with a wide range of electrodes, including carbon fiber [51], Au [117], Pt [112], or BDD [102]. Phosphate BGEs, however, can present challenges in CE separations because of their relatively high ionic strength, which leads to increased current and Joule heating, particularly at elevated separation voltages that may adversely affect separation efficiency [131]. For highly sensitive detection, etched‐joint decouplers [1], Nafion sleeves [56], or on‐capillary grounding is required. In addition, buffer composition and pH can influence the chemical stability of electroactive neurotransmitters, many of which are prone to so‐called auto‐oxidation in aqueous solution [132]. BGEs with higher ionic strength or inadequate control of pH and dissolved oxygen may accelerate this process, resulting in analyte degradation and compromised quantitative performance [133]. Careful BGE selection and optimization are therefore important not only for separation efficiency but also for minimizing analyte oxidation and preserving analytical reliability. LODs in phosphate systems span from nanomolar levels [74] into the low‐femtomole range when paired with suitable microelectrodes or redox‐cycling configurations.

2‐(*N*‐morpholino)ethanesulfonic acid (MES) BGEs form the second major category and have historically been the choice for high‐sensitivity detection in the pH 5.5–6.5 range [127]. Slightly acidic MES electrolytes have been widely used for the separation and amperometric detection of catecholamines such as DA and NE [62, 134]. These slightly acidic conditions minimize electrode fouling, suppress background current, and align well with the oxidation potential of catecholamines. Many of the best detection limits ever reported in CE–AD, down to attomole and even zeptomole levels, were achieved using 20–30 mM MES combined with carbon fiber microelectrodes in end‐column or minimally decoupled formats [62, 65]. Therefore, although MES is less frequently used overall than phosphate, it contributes disproportionately to the highest sensitivity results.

Borate BGEs are the third most widely used specific buffer class. They dominate high‐pH separations (pH 8–10) and especially MEKC systems containing SDS or other surfactants [135]. Borate offers strong complexation with diols (e.g., catecholamines), improving selectivity and thus allowing efficient separation of charged and neutral neurotransmitter derivatives. While borate–SDS systems achieved excellent separation selectivity and nanomolar detection limits, the lowest overall LODs were not obtained in borate but rather in slightly acidic systems. At high pH, oxidation potentials decrease, but adsorption and capillary/electrode fouling become more problematic, necessitating robust decoupling or high‐surface‐area electrodes.

Other BGEs are comparatively rare. 3‐(Cyclohexylamino)‐1‐propanesulfonic acid (TES) [136], Tris [137], and lithium acetate [138] appear mainly in specialized applications such as single‐cell MEKC or nanoscale capillary formats, often combined with SDS or organic modifiers. 3‐(Cyclohexylamino)‐1‐propanesulfonic acid (CAPS) (1 case) is used when buffering at pH > 9 is required without phosphate interference. No purely acetate‐based buffers were identified in this dataset for neurotransmitter CE–AD, although acetate sometimes appears as an additive or in sample stacking conditions. Another BGE type includes NaOH or HClO_4_/LiOH composition.

## 7 Applications

Table 4 provides a comprehensive overview of all CE–AD application studies, summarizing analytes, sample matrices, buffer systems, detection conditions, and achieved LODs. These examples highlight how CE–AD has evolved from proof‐of‐concept NT assays to a versatile analytical platform capable of addressing chemically diverse analytes in complex biological matrices and pharmaceutical formulations. The applications reviewed span excreted human biofluids, isolated cells, biofluids, and tissues from model organisms, and pharmaceutical products, illustrating the adaptability of CE–AD to samples that are often available only in limited volumes and require high selectivity without extensive derivatization.

**TABLE 4:** Applications of CE–AD.

Analytes	Sample matrix	BGE (composition + pH)	Detection details	LOD	Reference
DA, NE, E	Human urine (5× diluted)	0.15 M MES buffer adjusted to pH 5.57 with sodium acetate (optimal), also tested: acetate buffer pH 5.59 and MES–NaOH pH 5.57	Parallel‐opposed dual‐electrode; redox cycling; on‐capillary + disk electrode; +0.6 / –0.5 V vs. Ag/AgCl	DA 0.41 nM; E 0.14 nM; NE 0.16 nM	[127]
DA, NE, E, 5‐HT, TA, TrA	Human urine (centrifuged + SPE cleanup)	BGE: 150 mM boric acid + 1 mM ascorbic acid, pH 10.33; Sample: acetate buffer, pH 3.6	Dynamic pH junction CE–AD; carbon disk electrode; 0.9 V vs. SCE	5.34–68.3 nM (e.g. DA 12.6 nM; NE 18.4 nM; E 16.5 nM; 5‐HT 5.34 nM)	[96]
DA, NE, E, 5‐HT	Human urine (100× diluted with water–ACN 10:90 v/v, no off‐line pretreatment)	160 mmol/L sodium phosphate buffer, pH 5.9	End‐column amperometric detection using carbon fiber microdisk array electrode	DA: 0.44 nM, NE: 0.6 nM, E: 0.52 nM, 5‐HT: 0.3 nM	[89]
DA, NE, E, DOPAC	Peripheral blood lymphocytes (multiple sclerosis patients)	25 mM MES, pH 5.65	End‐column CE–AD; carbon fiber microelectrode; 0.8 V vs. SCE; etched capillary tip	DA & NE: 0.13 fmol/mg; E: 0.37 fmol/mg; DOPAC: 0.11 fmol/mg	[145]
DA, 3,4‐dihydroxybenzyl amine (DHBA), Isoproterenol enantiomers	In vivo rat plasma (microdialysis sampling from Sprague–Dawley rats)	100 mM lithium acetate buffer, pH 4.75, containing 0.1 g/mL methyl‐β‐cyclodextrin (chiral selector)	End‐column CE–amperometric detection (CE–EC) using carbon fiber microelectrode	0.63 ng/mL (≈3.2 nM) for ISP using pH‐mediated stacking	[138]
DA, NE, E, 5‐HT (monoamine neurotransmitters); plus metabolites (DOPAC, homovanillic acid (HVA), vanillic acid (VMA), 5‐hydroxyindoleacetic acid (HIAA)	Rat caudate nucleus microdialysate	For neurotransmitters: 0.16 M Na_2_HPO_4_/KH_2_PO_4_ buffer, pH 6.5. For metabolites + neurotransmitters: 50 mM Na_2_HPO_4_/KH_2_PO_4_, pH 7.0	End‐column electrochemical detection using 300 µm carbon disk electrode in wall‐jet configuration; Ag/AgCl reference	DA: 100 nM; NE: 30 nM; E & 5‐HT: 100 nM	[92]
Dopamine (DA) (also 4‐methylcatechol as internal standard)	Rat brain microdialysate (1‐min collection)	20 mM phosphate buffer, pH 7.4; with 2 mM EDTA added when analyzing aCSF/microdialysate to eliminate metal ion interference	Integrated etched end‐column decoupler CE–EC; carbon fiber microelectrode (radius 3.5 µm, 200–300 µm exposed), +600 mV vs. Ag/AgCl	∼5 nM DA (concentration), ∼38 amol injected (from 5 nM standard)	[86]
LDME (levodopa methyl ester), L‐DOPA, DA	Rat serum (after intravenous administration of LDME or L‐DOPA)	50 mM phosphate, pH 7.0	End‐column CE–AD using 300 µm carbon disk electrode; detection potential +1.00 V vs. Ag/AgCl	LDME: 14.6 ng/mL; L‐DOPA: 98.0 ng/mL; DA: 9.7 ng/mL	[139]
Melatonin (MT), serotonin (5‐HT), tryptophan (Trp), 5‐hydroxytryptophan (5‐HTrp)	Rat pineal gland extract (tiny tissue, ∼1 mg; homogenized in perchloric acid)	0.20 mol/L phosphate buffer (NaH_2_PO_4_/Na_2_HPO_4_), pH 8.0	End‐column CE–EC with 300 µm carbon disk electrode (wall‐jet); detection potential +0.90 V vs. SCE	5‐HT: 0.03 µM; MT: 0.12 µM; Trp: 0.13 µM; 5‐HTrp: 0.10 µM	[137]
Norepinephrine (NE, endogenous)	Rat heart tissue homogenates (after perchloric acid extraction + SPE cleanup	250 mM boric acid/KOH buffer, pH 8.8	End‐column amperometric detection; boron‐doped diamond microelectrode, +0.86 V vs. Ag/AgCl	0.034 µg/g tissue (ventricles and septum), 0.22 µg/g tissue (atria)	[105]
DA, 5‐HT, Adenosine	Rat brain slice tissue punches (caudate–putamen, prefrontal cortex)	150 mM NaH_2_PO_4_ + 1 mM β‐cyclodextrin, pH 4.0	End‐column CE with FSCV; 34.5 µm carbon‐fiber microelectrode, scan −0.4 to +1.5 V at 400 V/s	DA: 5 ± 3 nM; 5‐HT: 10 ± 3 nM; Adenosine: 50 ± 20 nM	[141]
L‐DOPA, DA, NE, E, OA, TA, 5‐HT (standards); L‐DOPA, DA, TA, 5‐HT quantified in fly homogenates	Drosophila melanogaster head and body homogenates (male and female, wild‐type & transgenic)	10 mM TES buffer + 30 mM SDS + 2% 1‐propanol, pH 7.1	End‐column MEKC–EC; 5 µm Nafion‐coated carbon fiber microelectrode; +0.70 to +0.75 V vs. Ag/AgCl	L‐DOPA: 4 amol; Epinephrine (E): 20 amol (others in same range)	[146]
DA, NE, E, OA, 5‐HT, L‐DOPA, DOPAC,	*Drosophila melanogaster* head homogenates (wild‐type and mutant); perchloric acid extraction	25 mM borate + 50 mM SDS + 2% 1‐propanol, pH 9.5	MEKC–electrochemical detection; 5 µm carbon fiber microelectrode at +0.70 to +0.75 V vs. Ag/AgCl; 13 µm i	OA: 3.4 amol; L‐DOPA: 112 amol; others between these values	[135]
L‐DOPA, naOA, naDA, naTA, na5‐HT, OA, DA, TA, 5‐HT	Single *Drosophila melanogaster* head homogenates (250 nL homogenization buffer)	10 mM TES + 30 mM SDS + 2% 1‐propanol, pH 7.09	5 µm carbon fiber microelectrode, +0.70 to +0.75 V vs. Ag/AgCl	∼4 amol (L‐DOPA), ∼20 amol (E); single fly data mainly reported in fmol/picomole quantities (not concentration‐based)	[147]
DA, 5‐HT, OA, TA	Single *Drosophila* larval central nervous system (CNS), homogenized in perchloric acid + acetonitrile	200 mM phosphate buffer + 1 mM tetraborate, pH 4.5	End‐column CE with FSCV carbon‐fiber microelectrode (34.5 µm); scan −0.4 to +1.3 V at 400 V/s	DA & 5‐HT: 1 nM; TA: 2.5 nM; OA: 4 nM (with field‐amplified sample stacking)	[97]
DA	Single dopamine neuron from *Planorbis corneus* (snail brain), injected intact into a capillary and lysed on‐column	25 mM MES buffer, pH 5.65	Scanning electrochemical detection (voltammetric CE–EC)**;** carbon fiber microelectrode (5 µm) inserted into HF‐etched capillary tip; potential scanned −0.2 to +1.0 V vs. Ag/AgCl	∼400–460 fmol total DA per cell (∼300–850 µM in cell volume); no molar LOD given	[87]
NE, E (native); DA, L‐DOPA after precursor incubation	Single bovine adrenal chromaffin cells; also, the extracellular medium during nicotine‐induced release	10 mM TES + 10 mM SDS, pH 7.0	MEKC–EC, carbon fiber microelectrode (5 µm), +0.65 V vs. Ag/AgCl; end‐column	∼1–5 fmol NE/E in single cells; DA after L‐DOPA: ∼0.3–0.5 fmol; extracellular NE/E release: ∼6–8 fmol	[148]
DA (intracellular)	Cytoplasm of single PC12 mammalian cells (rat pheochromocytoma), sampled via nanocapillary injection	50 mM TES + 2% 1‐propanol, pH 7.2	End‐column amperometric detection in 770 nm i.d. capillary; flame‐etched carbon fiber microelectrode (5 µm), +0.70 V vs. Ag/AgCl; etched capillary tip for alignment (nanometer‐scale CE–EC)	∼400 ± 100 zmol DA (standard solution LOD); intracellular cytoplasmic dopamine: 240 ± 60 mM	[46]
Him	Single rat peritoneal mast cells (whole‐cell injection, lysed on‐capillary)	15.6 mM NaH_2_PO_4_/24.4 mM Na_2_HPO_4_, pH 7.0	End‐column CE–AD with carbon fiber microdisk bundle electrode (∼30 fibers, 6 µm diameter each); +1.30 V vs. SC	∼96 fmol histamine per cell (*n* = 9)	[142]
L‐DOPA, Carbidopa	Pharmaceutical tablets (Sinemet) — dissolved, filtered solutions	80 mM phosphate buffer (NaH_2_PO_4_/Na_2_HPO_4_), pH 7.0	End‐column CE–AD; carbon disk electrode (500 µm pencil lead); +0.90 V vs. Ag/AgCl (3 M KCl); platinum auxiliary electrode	L‐DOPA: 0.6 µg/mL (∼3.0 µM); Carbidopa: 0.3 µg/mL (∼1.5 µM)	[143]
L‐DOPA, Benserazide (BS), Ro‐04‐1419 (impurity)	Pharmaceutical formulations (co‐beneldopa tablets and granules)	40 mM phosphate buffer, pH 5.3	End‐column CE–AD; carbon disk electrode (300 µm), +0.95 V vs. Ag/AgCl	L‐DOPA: 0.38 µg/mL (∼2 µM); BS: 0.02 µg/mL (∼0.06 µM); Ro‐04‐1419: 0.004 µg/mL (∼0.03 µM)	[144]

### 7.1 Human‐Derived Samples

The analysis of human‐derived matrices represents one of the important application areas of CE–AD for clinical diagnostics. The studies reviewed below focus on two representative types of human samples: excreted biofluids, exemplified by urine, and isolated cells, particularly peripheral blood lymphocytes. Catecholamines and related indoleamines in urine serve as established biomarkers for a range of endocrine, neurological, and psychiatric disorders [149], while peripheral blood lymphocytes are of particular interest, as they are readily accessible and play a role in neuroimmune communication [150].

#### 7.1.1 Human Urine

Urine is a readily accessible, noninvasively collected biofluid that reflects systemic catecholamine and indoleamine metabolism and is routinely used in clinical diagnostics, particularly for disorders associated with altered monoamine excretion. For instance, pheochromocytomas and paragangliomas are catecholamine‐producing neuroendocrine tumors that cause excessive sympathetic activation. Their diagnosis relies heavily on measuring catecholamines and their metabolites in urine [151]. CE–AD quantification of MNTs in human urine offers a direct, rapid, and cost‐effective solution to MNT analysis comparable to conventional HPLC–ED methods. In particular, 24 h urine collections smooth pulsatile secretion (particularly relevant for catecholamine tumors and serotonin secretion) and are thus often used for NT analysis [152] in all reported publications.

Direct analysis of 24 h urine samples by Chen et al. [127] demonstrated that DA, E, and NE could be quantified at nanomolar levels by using a parallel‐opposed dual‐electrode detector. The method employed 150 mM MES/sodium acetate BGE (pH 5.6) and yielded well‐resolved peaks within 12 min, with sensitivity sufficient for physiological catecholamine concentrations (LODs were in the range of 0.14 to 0.64 nM). The high achieved sensitivity was due to the redox cycling on the dual electrode design and allowed direct analysis in fivefold diluted urine. Since 24‐hour urine samples collected from healthy adults have the known reference values of epinephrine and norepinephrine at 10 µg (55 nmole) and 100 µg (591 nmole), respectively, the sensitivity of the developed methods was sufficient to distinguish the healthy and cancer patients.

**FIGURE 6:**
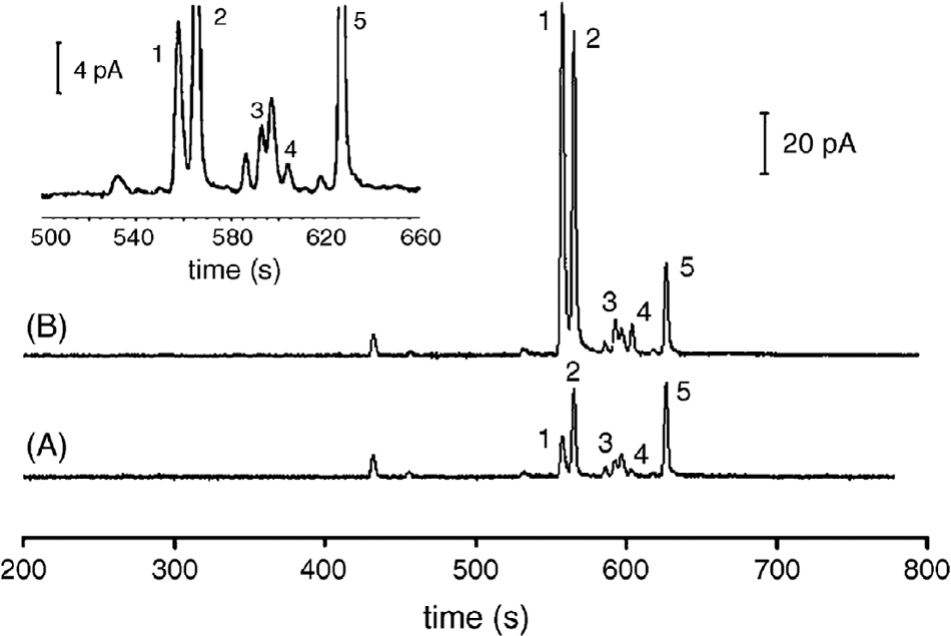
Electropherograms of a urine sample (A) from a healthy man and a spiked sample (B) under optimum conditions. The inset shows an expanded portion of the curve (A) electropherogram. Capillary column, 66 cm × 25 µm id; separation voltage, 20 kV; BGE, 160 mmol/L salt phosphate (pH 5.9). Peak identification: 1, 5‐HT; 2, DA; 3, NE; 4, E; 5, IP. *Source*: Reproduced from *Journal of Chromatography B* [89] with permission from Elsevier.

Tang et al. [96] improved the analysis of urine by including up to six biogenic amines (DA, E, NE, 5‐HT, TA, and TrA). The authors introduced a dynamic pH junction on‐line preconcentration and used a carbon disk electrode for detection. A large acidic sample plug (150 mM acetate buffer, pH 3.6) was electrokinetically injected into a capillary filled with basic boric acid BGE (150 mM, pH 10.33). The sharp pH discontinuity at the boundary created a moving focusing zone, leading to approximately 100‐fold signal enhancement relative to conventional CE–AD. All analytes were separated within 20 min with theoretical efficiencies exceeding 10^5^ plates, and LODs ranged from 5.3 to 68 nM. Importantly, this dynamic pH‐junction approach required only a mild solid‐phase extraction step (alumina B) and achieved excellent recoveries without derivatization. Aiming at further improvement of concentration sensitivity, Weng et al. [89] developed a FASS protocol coupled with CE–AD for DA, NE, E, and 5‐HT in urine. The use of a carbon‐fiber microdisk array electrode (ca. 60 fibers, 6 µm diameter) and phosphate BGE (160 mM, pH 5.9) provided enhanced surface area and stable electrochemical performance. The FASS procedure exploited conductivity differences between the sample and BGE (urine was diluted and dissolved in 10/90 H_2_O–ACN), resulting in an effective sensitivity gain of ca. 50‐fold in urine (5000‐fold in standard solution). LODs reached 0.3–0.6 nM, linearity spanned from 1 to 25 nM, and the analysis required no off‐line enrichment, representing one of the most sensitive CE–AD urine assays reported to date. The separation of NTs in a healthy subject (Figure 6A) and the same spiked sample (Figure 6B) is shown below.

These reports establish CE–AD as a capable technique for routine urinary NT analysis. Advances in on‐line stacking (FASS) and dynamic pH junction focusing have overcome some of the sensitivity limitations, reaching sub‐nanomolar levels, but only in model solutions. The developed methods are nevertheless suitable for clinical diagnostics such as pheochromocytoma screening. Matrix effects from high salt content in urine still require attention; controlled dilution or solid‐phase cleanup remains critical for reproducibility [89, 96]. From this angle, the method by Chen [127] using only a fivefold dilution with no other sample pretreatment and parallel opposed dual‐electrode redox cycling seems to be the most promising one for urine sample analysis.

#### 7.1.2 Human Peripheral Blood Lymphocytes

In a complementary “cells‐as‐samples” direction alongside urine assays for human clinical neurochemistry, particularly when the biological question concerns intracellular catecholamine pools in circulating immune cells, CE–AD offers clinical insight complementary to biofluid assays. Multiple sclerosis (MS) is an inflammatory demyelinating disease of the central nervous system (CNS). The deactivation of the immune system after an MS relapse could be mediated by catecholamines. Accordingly, the intracellular levels of catecholamines in relapsing MS patients and in first‐attack MS patients were compared by Rajda et al. [145]. The authors quantified intracellular DA, NE, E, and metabolites from peripheral blood lymphocytes of 58 MS patients versus 19 controls using CE with end‐column carbon‐fiber amperometric detection (0.8 V vs. SCE), electrokinetic injection, and a low‐pH 25 mM MES buffer (pH 5.65). They reported elevated epinephrine in first‐attack MS patients and reduced norepinephrine in relapsing‐remission MS patients, supporting a role for catecholaminergic immunomodulation.

### 7.2 Model Organism–Derived Samples

Model organisms have played a central role in the development and validation of CE–AD for neurochemical analysis, providing experimentally accessible systems in which sampling conditions, biological variability, and analytical performance can be systematically controlled. Applications in this area primarily target biofluids, tissues, and isolated cells, often under conditions where sample volumes are extremely limited. The use of animal and invertebrate models has enabled investigations of neurotransmitter dynamics, pharmacokinetics, and tissue‐specific neurochemistry that are difficult or impossible to perform in humans. The following subsections summarize CE–AD applications in rodent and invertebrate systems, highlighting their complementary roles in neurochemical research.

#### 7.2.1 Rodent Biofluids

A major focus has been the analysis of rodent blood and brain microdialysates, where CE–AD enables quantification of catecholamines and related compounds in minute, time‐resolved samples without perturbing extracellular neurochemistry. CE–AD can achieve the sensitivity required for low‐nanomolar NT concentrations while supporting short sampling intervals that can capture rapid neurochemical changes induced by drugs or electrical stimulation, as will be discussed below. Beyond microdialysates, CE–AD has also been applied to rodent serum samples.

The pioneering work of Hadwiger et al. [138] first demonstrated the feasibility of on‐line CE–AD monitoring of catecholamines in blood microdialysates, combining in vivo sampling with electrophoretic separation. Microdialysis probes were implanted intravenously in Sprague–Dawley rats, enabling continuous sampling of the free plasma fraction of isoproterenol with a temporal resolution of 1 min. Although not specifically applied to NT analysis, in this article, DA and DHBA were applied as an internal standard and perfusion fluid calibrator. The authors were able to simultaneously monitor the elimination kinetics of the individual enantiomers of isoproterenol directly in living animals after administration of the racemic mixture. As a proof of concept, Jin et al. [92] coupled CE–AD with microdialysis for the determination of MNTs and their metabolites directly from rat caudate‐nucleus dialysates. The caudate nucleus typically contains high levels of monoamine neurotransmitters and is commonly used for microdialysis experiments in rats to study neurotransmitter release and metabolism. Using a 300 µm carbon‐disk electrode operated in a wall‐jet configuration and a 50 mM phosphate BGE (pH 7.0), the authors achieved clear separation of eight compounds, including DA, NE, E, 5‐HT, and their acidic metabolites (DOPAC, HVA, VMA, 5‐HIAA) with LODs of 100 nM for individual neurotransmitters. The system enabled monitoring of a 25 µL dialysate sample showing concentrations of NT in the range of 10 to 100 nM; however, their metabolites were below the method´s LOD. To advance the application area further, Qian et al. [86] analyzed DA, DOPAC, and 5‐HIAA from rat brain microdialysate with LODs near 1 nM. This sensitivity was achieved using an integrated etched decoupler that efficiently dissipated separation current and allowed stable amperometric detection. With a similar temporal resolution as previous works, the presented CE–EC method could monitor physiologically relevant changes in extracellular DA, including increases caused by pharmacological inhibition of DA uptake and transient increases triggered by electrical stimulation of dopaminergic neurons. This demonstrates the method's capability for real‐time monitoring of neurotransmitter dynamics in vivo.

Going from off‐line collection and analysis of microdialysis samples, Zhou et al. [136] developed a fully on‐line microdialysis–CE–AD system capable of in vivo microdialysis sampling with CE–AD, representing an important advancement in real‐time bioanalytical monitoring. The on‐line microdialysis–CE–AD system with a 33 µm CFE at +0.8 V versus Ag/AgCl and a dual‐decoupler configuration was used to maintain current stability. Catecholamine standards (DA, hydroquinone, DOPAC) were separated within 7 min in a 50 mM TES BGE (pH 7.4). The work illustrated CE–AD's capacity for pharmacokinetic profiling in vivo, with LODs below 100 nM and consistent peak shapes even with high‐salt physiological dialysates. Wang et al. [139] focused on rat serum analysis of levodopa methyl ester (LDME), L‐DOPA, and DA, developing a CE–AD protocol compatible with clinical pharmacokinetic samples. A 50 µm id capillary and 50 mM phosphate BGE (pH 7.0) were used with electrokinetic injection, and a carbon disk in an end‐column geometry. With low LODs (57 nM for DA), the method demonstrated clear conversion of LDME to L‐DOPA and DA following oral administration and allowed simultaneous monitoring of the prodrug and its metabolic conversion. The results showed rapid hydrolysis of LDME in vivo and measurable serum levels of L‐DOPA and dopamine at different time points after administration.

While some of the early studies only provided proof of concept for in‐vivo microdialysis of various rodent biofluids, later work has demonstrated that such sampling can monitor for instance changes in NT concentrations after drug administration or electric stimulus, or even monitor the conversion of the prodrug (LDME) to DA, paving the way to use these techniques and methods to study neurotransmitter dynamics, drug metabolism, and therapeutic mechanisms in disorders involving catecholaminergic dysfunction.

#### 7.2.2 Rodent Tissues

Beyond blood–brain microdialysates and serum analysis, CE–AD has also been employed for the direct analysis of solid tissues, such as minute homogenate or microdissected regions. In here, the unprecedented capacity of CE to analyze extremely small samples (less than 1 µL or 1 mg of tissue) comes as a clear advantage, compared with conventional HPLC analysis. Cheng et al. [140] pioneered the application of CE–AD to the rat pineal gland, which is only about 1 µL in volume and normally makes the analysis very challenging. The pineal gland is a tiny endocrine organ in the brain that produces melatonin and regulates circadian rhythms. It is biologically important for sleep regulation, neuroendocrine signalling, and antioxidant defense, and analytically important because it contains key indole neurotransmitter metabolites that can be measured to study neurochemical pathways. By developing a novel microinjector enabling electrokinetic injection of sub‐microliter tissue extracts, CE analysis could be performed without excessive dilution. The system employed a 300 µm carbon disk electrode arranged in a wall‐jet configuration, operated at +0.9 V vs. SCE, and used 200 mM phosphate BGE (pH 8.0). 5‐HT was fully resolved from melatonin, 5‐hydroxytryptophan, and tryptophan within 15 min, with LODs of 0.03–0.13 µM. The system thus allowed quantitative analysis of melatonin biosynthesis intermediates directly in pineal tissue.

Zhang et al. [137] reported the direct determination of dopamine in single cultured rat pheochromocytoma (PC12) cells. PC12 cells are widely used as a model of sympathetic neurons, meaning that the ability to measure dopamine in single PC12 cells provides a valuable analytical approach for studying neurotransmitter storage, release, and neuronal physiology at the cellular level. The separation was carried out in a 10 mM Tris BGE (pH 6.2), and AD was performed using a single carbon‐fiber microelectrode positioned end‐column in a two‐electrode configuration. The method enabled quantification of DA at the femtomole level, with measured DA contents in individual PC12 cells ranging from 0.29 to 1.28 fmol (mean 0.61 ± 0.30 fmol, *n* = 10 cells).

In yet another account where sample size was limited, Fang et al. [141] introduced a hybrid CE–FSCV system capable of quantifying DA and 5‐HT from nanoliter‐scale rat brain tissue punches with high sensitivity (LOD values of 5 nM for DA and 10 nM for 5‐HT were obtained) and required as little as 7 µg of tissue. This represents three orders of magnitude smaller sample volumes than typical HPLC‐based tissue analysis, demonstrating the high mass sensitivity of the CE‐based method. When applied to successive striatal slices, the authors found higher DA and lower adenosine content in anterior regions and a strong correlation (*R* ^2^ = 0.71) between DA tissue content and stimulated release measured in situ. The developed system provided an important tool for studying the neurochemical organization of specific brain regions. Such measurements allow researchers to correlate local neurotransmitter storage with functional release measurements, improving our understanding of neuronal signaling and the neurochemical basis of brain function.

Complementary to these central‐nervous‐system studies, Novotny et al. [105] applied CE with end‐column AD to quantify endogenous NE in anatomically distinct regions of the rat heart, including the left and right atria, left and right ventricles, and the ventricular septum. Separation was performed in a 250 mM boric acid/KOH BGE (pH 8.8), exploiting borate complexation to impart a net negative charge to NE, while electrokinetic injection introduced nanoliter‐scale sample volumes. AD was carried out using an end‐column BDD microelectrode, which provided a low and stable background current (≤0.65 pA peak‐to‐peak noise) and excellent resistance to electrode fouling during repeated injections of tissue homogenates. Regional measurements across atrial and ventricular tissue highlighted pronounced differences in NE distribution, illustrating CE–AD's potential for comparative biochemical mapping of peripheral tissues.

CE–AD enables direct analysis of extremely small solid tissue samples (<1 µL or <1 mg), for instance, rat pineal glands, providing a major advantage over HPLC for studying localized neurochemistry in rodent tissues. At an even smaller sample scale, DA was quantified in single PC12 cells using end‐column carbon fiber detection, reaching femtomole levels. Complementary studies using BDD electrodes in heart tissue demonstrated regional differences in NE distribution, highlighting CE–AD as a powerful tool for spatially resolved neurochemical mapping in both central and peripheral tissues.

#### 7.2.3 Invertebrate Tissues

*Drosophila melanogaster* (fruit fly) serves as a powerful invertebrate model for neurochemical studies because many monoaminergic signaling pathways, including those of DA and 5‐HT, are evolutionarily conserved with invertebrates. In addition, its compact nervous system and extensive genetic toolkit make it highly tractable for detailed studies of neurotransmission [153]. Profiling of biogenic amines and their metabolites in exceptionally small biological samples, ranging from pooled fly tissues to individual nervous systems, has been enabled by CE–AD. These studies have demonstrated that neurochemical mechanisms in invertebrates closely parallel those in mammals, supporting the translational relevance of fly‐based models. As a result, CE–AD applications in invertebrates have contributed significantly to understanding neurotransmission, neuromodulation, and genetic influences on neurochemistry.

Two early studies illustrate how CE–AD, operated in MEKC mode, enables quantitative analysis of biogenic amines and their metabolites in minute fly‐derived samples. Ream et al. [146] demonstrated MEKC–AD for the analysis of electroactive biogenic amines in pooled *Drosophila* head and body homogenates, using a single carbon‐fiber microelectrode positioned end‐column and a TES/SDS BGE (10 mM TES, 30 mM SDS, 2% 1‐propanol, pH 7.1). The method resolved key monoamines relevant to fly neurochemistry, including L‐DOPA, DA, TA, OA, and 5‐HT, and enabled quantitative comparisons between wild‐type and transgenic flies with functionally silenced monoaminergic neurons, achieving attomole‐level mass detection limits. The analysis revealed distinct neurochemical profiles between the fly head and body tissues, i.e., Tyr and 5‐HT were much more abundant in the head, while DA was more abundant in the body, likely due to its role in cuticle formation and sclerotization in insects. The study also analyzed transgenic flies lacking functional DA/5‐HT neurons, finding that DA was no longer detectable in mutant flies and 5‐HT levels were drastically reduced. On the other hand, Tyr remained unchanged, consistent with its synthesis in different neuronal populations. Paxon et al. [135] extended this approach by optimizing borate‐based MEKC–AD for the simultaneous separation of biogenic amines and their metabolites, totaling 14 analytes, including *N*‐acetylated products (e.g., *N‐*acetyldopamine, *N‐*acetylserotonin, *N*‐acetyloctopamine) and oxidative metabolites (e.g., DOPAC, HVA, 5‐HIAA), in *Drosophila* head homogenates. Using narrow id capillaries and a 25 mM borate/50 mM SDS BGE (pH 9.5), the study achieved reproducible, high‐efficiency separations of 14 analytes within 20 min, establishing MEKC–AD as a robust platform for probing monoamine metabolism and genetic perturbations in the fly nervous system. One of the most important biological conclusions of this work was that *N*‐acetylated monoamine metabolites were abundant in *Drosophila* samples. On the other hand, classical oxidative deamination products such as DOPAC, HVA, and 5‐HIAA were largely absent or very low. This suggests that monoamine metabolism in insects occurs primarily through *N*‐acetylation rather than monoamine‐oxidase pathways typical of mammals.

Powell et al. [147] advanced CE–AD to the single‐fly level, quantifying neurotransmitter variability among individual fly heads rather than in pooled populations as done in previous articles. The homogenates having a volume of 100–250 nL revealed inter‐individual neurochemical variability in DA, 5‐HT, TA, na5‐HT, naTA, and OA. For instance, monoamine levels varied significantly between individual flies, even when they originated from the same genetic strain and environmental conditions. The study also confirmed the previous findings by Paxon et al. [135] on the abundance of *N*‐acetylated metabolites. Finally, Fang et al. [97] improved the sensitivity and selectivity by integrating FSCV as the detector after CE separation, in the analysis of biogenic amines from a single *Drosophila* larval brain. The method combined FASS with end‐column FSCV, using an etched capillary and a 34 µm carbon‐fiber disk electrode. LODs reached 1 nM for DA and 5‐HT, 2.5 nM for TA, and 4 nM for OA, enabling full neurotransmitter quantification in one larval brain (ca. 8 nL). The method combines electrophoretic separation with electrochemical detection, which provides both migration time and voltammetric fingerprints, enabling improved chemical identification compared with conventional amperometric detection, and can detect neurochemical changes caused by genetic mutations or pharmacological treatments.

The above studies illustrate the evolution of CE–AD from population‐level to single‐organism analysis, establishing it as a precise, label‐free platform for functional neurochemical phenotyping in small genetic models. The analyses showed that monoamine metabolism in insects is dominated by *N*‐acetylated metabolites (e.g., naDA, na5‐HT), highlighting fundamental differences from mammalian monoamine degradation pathways. Furthermore, the ability to detect strain‐dependent variability and changes induced by genetic mutations or pharmacological inhibition demonstrated that these analytical approaches can directly link neurochemical levels with genetic regulation and neuronal function in *Drosophila*.

#### 7.2.4 Single Cell Analysis

Single‐cell analysis has emerged as a crucial approach for understanding neurochemical communication within complex biological systems, where population‐averaged measurements often obscure cell‐to‐cell heterogeneity. Several studies demonstrated that CE–AD could interrogate neurochemical contents within single neurons, chromaffin cells, and other excitable or secretory cell types. In the nervous system, individual neurons and secretory cells can differ markedly in neurotransmitter content, storage, and release dynamics. CE‐based methods could resolve and quantify catecholamines, indoleamines, and Him from individual neurons, chromaffin cells, and mast cells, revealing distinct intracellular storage pools and release mechanisms. These advances established single‐cell CE–AD as a key tool for elucidating fundamental aspects of neurotransmission, cellular heterogeneity, and stimulus‐dependent neurochemical regulation that are inaccessible at the tissue or biofluid level.

A pivotal biological application was carried out by Swanek et al. [87], who used CE–AD to identify multiple intracellular DA compartments within a single giant neuron of the pond snail *Planorbis corneus*. Microinjection sampling, coupled with CE–AD separation, and scanning AD revealed at least two distinct DA pools differing in oxidation potential and concentration. Scanning electrochemical detection provided voltammograms for each peak during electrophoretic separation, allowing identification and confirming that both peaks correspond to DA rather than different compounds. The results thus provided direct experimental evidence that DA is stored in multiple vesicular compartments within a single neuron, offering new insight into neurotransmitter storage and release mechanisms in the nervous system.

Later, Suljak et al. [148] used MEKC–AD to analyze catecholamine content in single bovine chromaffin cells, which serve as a model for neuronal catecholamine storage and release. The study achieved fmol LODs for NE and DA and revealed large variability in catecholamine content between individual adrenal cells, with some cells containing mostly E while others contained significant NE. This observation contradicts earlier histochemical models suggesting that individual chromaffin cells store only one catecholamine type. The method was also used to monitor exocytotic release of catecholamines from individual chromaffin cells stimulated with nicotine. The measured extracellular release corresponded to the release of catecholamines from approximately 3000 vesicles, demonstrating that the technique can quantify neurotransmitter release dynamics at the single‐cell level.

Woods et al. [46] further miniaturized the CE–AD system by using nanometer‐scale capillaries (770 nm id). Such extremely small capillaries dramatically reduce the injected sample volume and are therefore suitable for analysis of microscopic biological environments such as single cells. Thus, the cytoplasmic contents of single PC12 cells were analyzed. DA was detected from less than 10% of the cellular volume with minimal perturbation to cell integrity, demonstrating the attoliter sampling scale. This work represents an important step toward analytical techniques capable of probing neurochemical processes at the single‐cell and subcellular level.

While most single‐cell CE–AD studies focused on catecholaminergic neurons, Weng et al. [142] broadened the scope by quantifying Him release from single rat peritoneal mast cells. Using a carbon fiber micro disc bundle electrode and phosphate BGE (pH 7.0), they directly analyzed the contents of individual cells lysed inside the capillary. The average Him content (96 fmol per cell) agreed with literature values, verifying CE–AD's accuracy for nonneuronal secretory systems. A significant finding was the large variability in Him content between individual mast cells, where the measured amounts differed by nearly an order of magnitude. This demonstrates the importance of single‐cell analysis rather than population‐averaged measurements.

CE–AD is thus capable of resolving neurotransmitter heterogeneity, secretion dynamics, and subcellular compartmentation within single cells and is uniquely suited for studying cellular communication at the molecular level. Measurements in individual cells revealed typical intracellular levels such as ca. 460 fmol DA in a single neuron, ca. 58 fmol total catecholamines in bovine chromaffin cells, and ca. 96 fmol Him in individual rat mast cells, highlighting the extremely small quantities involved in cellular neurochemistry. Furthermore, the studies consistently showed large cell‐to‐cell variability in analyte concentrations, demonstrating that population‐averaged measurements can obscure significant biological heterogeneity.

### 7.3 Pharmaceutical Formulations

Although most CE–AD applications have focused on biological matrices, the technique has also found utility in the quality control and formulation analysis of pharmaceutical products, particularly for catecholaminergic drugs used in Parkinson's disease therapy. Zhang et al. [143] reported one of the earliest CE–AD determinations of L‐DOPA and carbidopa in pharmaceutical tablets, where sensitivity is not critical. A carbon disk electrode operated at +0.9 V (vs. Ag/AgCl) enabled simultaneous separation of both analytes within 6 min, with moderate LODs of 0.4 µM for L‐DOPA and 0.7 µM for carbidopa. The assay showed excellent linearity and recovery between 98%–102%, demonstrating the potential of CE–AD for routine pharmaceutical analysis without derivatization or complex pretreatment. Further refinement was achieved by Wang et al. [144], who developed a CE–AD protocol for the quantitation of L‐DOPA, benserazide, and its process impurity Ro‐04‐1419 in co‐beneldopa pharmaceutical formulations delivering highly reproducible results with LODs around 0.1 µM and RSD < 3%.

## 8 Future Trends, Perspective, and Conclusions

Although CE–AD has been successfully applied to human urine and, to a lesser extent, blood and lymphocytes, its use in human clinical diagnostics remains limited to a few clinical examples. Most studies still focus on animal models, animal brain microdialysates, or tissue extracts, while truly noninvasive human biofluids remain largely unexplored. Future progress should therefore expand CE–AD toward alternative sample types such as sweat, saliva, and exhaled breath condensate, which offer painless collection, minimal ethical restrictions, and the potential for continuous or wearable monitoring. However, these matrices contain neurotransmitters at extremely low concentrations and are often rich in salts, proteins, or surfactants, necessitating improved preconcentration strategies, antifouling electrode surfaces, and robust on‐line sample handling.

Advances in microfabrication, disposable electrode–capillary modules, and microfluidic integration are expected to enhance reproducibility and portability, enabling point‐of‐care or bedside neurochemical measurements. The combination of CE–AD with redox‐cycling electrodes, field‐amplified stacking, or dynamic pH junctions will likely further decrease LODs, while hybrid electrochemical–mass spectrometric or electrochemical–spectroscopic schemes could broaden the analyte range. Ultimately, the transition of CE–AD from a laboratory tool to a clinically relevant technique will depend on improving robustness, automation, and interfacing with minimally invasive or wearable sampling platforms. CE–AD thus remains a promising yet evolving tool in neurochemical analysis.

## Author Contributions

**Petr Kubáň**: Conceptualization, supervision, project administration, funding acquisition, writing – original manuscript. **Jiří Volánek**: Conceptualization, writing – review and editing**. Nguyen Thi Thu Trang**: Writing – review and editing. **Tran Dai Lam**: conceptualization, writing – review and editing.

## AI Usage Statement

An AI‐based large language model (ChatGPT) was used solely as an auxiliary tool during the preparation of this review to assist with language editing and stylistic refinement. No AI system was used to generate original experimental data, figures, results, or scientific claims. All factual content, interpretations, and references were independently verified by the authors against the primary literature. The authors retain full responsibility for the scientific accuracy and originality of the manuscript.

## Conflicts of Interest

The authors declare no conflicts of interest.

## Supporting information



Supporting Information data of this article can be found online at https://doi.org/10.1002/elps.70099. **Supporting File**: elps70099‐sup‐0001‐SuppMat.docx.

## Data Availability

Data sharing is not applicable to this article as no datasets were generated or analyzed during the current study.
