# Supplementary material for: Capillary Electrophoresis With Amperometric Detection for Neurotransmitter Analysis: Principles, Electrode Materials, Methodologies, and Applications
Source: Electrophoresis. 2026 May 2;47(6):450–78. doi: 10.1002/elps.70099 (PMC13273354; doi:10.1002/elps.70099)
Supplement: Supplementary file 1 — Supporting Information data of this article can be found online at https://doi.org/10.1002/elps.70099. Supporting File: elps70099‐sup‐0001‐SuppMat.docx. [file ELPS-47--s001.docx]

**SUPPLEMENTARY MATERIAL**

**Capillary electrophoresis with amperometric detection for neurotransmitter analysis: Principles, electrode materials, methodologies, and applications**

Petr Kubáň^1^, Jiří Volánek^1,2^, Nguyen Thi Thu Trang^3^, Tran Dai Lam^3^

*^1^Department of Bioanalytical Instrumentation, Institute of Analytical Chemistry of the Czech Academy of Sciences, v.v.i., Veveří 97, 60200 Brno, Czech Republic.*

*^2^Department of Chemistry, Faculty of Science, Masaryk University, Kamenice 5, 625 00 Brno, Czech Republic*

*^3^Institute of Materials Science, Vietnam Academy of Science and Technology,* 18 Hoang Quoc Việt, Nghia Do, Ha Noi, Vietnam

**Corresponding author: Assoc. Prof. Petr Kubáň, email:* [*petr.kuban@iach.cz*](mailto:petr.kuban@iach.cz)*, Tel: +420 532290142,* *Fax: +420 532 290 182. Permanent address: Institute of Analytical Chemistry of the Czech Academy of Sciences, Veveří 97, 60200, Brno, Czech Republic.*

## **Table S1.Oxidation Potentials of Selected Biomolecules (pH ~7.4, 25 °C vs Ag/AgCl)**

___________________________________________________________________________

| **Compound** | **Experimental E°′** (V) *[method; electrode]* | **Predicted E°′** (V) *[source]* |
| --- | --- | --- |
| **Dopamine** | ~0.11 V *[CV; Au nano-array electrode]*[[1]](https://www.nature.com/articles/s41598-018-32477-0?error=cookies_not_supported&code=5d465744-c867-42af-b938-26c09bc41a48#:~:text=concentrations.%20No%20dopamine,standard%20deviation%20of%20the%20response). Peak anodic/cathodic ~+0.14/+0.09 V in PBS, giving formal E°′ ≈ +0.12 V. | ~0.00 V *[DFT-calculated; vs SHE→Ag/AgCl]*[[2]](https://link.springer.com/article/10.1134/S0036024414070280#:~:text=theoretical%20calculations,Asp%2FDAquinone). *Calculated standard E° ≈0.63 V vs SHE (pH0) corresponds to ~0 V at pH7.4*. |
| **Norepinephrine** | ~0.40 V *[CV; bare glassy C]*[[3]](https://www.mdpi.com/2227-9040/9/1/14#:~:text=Nanomaterials%20Based%20Electrochemical%20Sensors%20for,mV%20on%20the%20unmodified). (Oxidation peak on unmodified GC ≈+0.4 V vs Ag/AgCl, lowered to +0.18 V with catalysis). | ~0.20 V *[est. via catecholamine trends]*[[4]](https://pmc.ncbi.nlm.nih.gov/articles/PMC8822484/#:~:text=,For). *(Catecholamine E°′ in water ≈0.4 V vs NHE ≈0.2 V vs Ag/AgCl)*. |
| **Epinephrine** | ~0.24 V *[CV; AuNP-modified GC]*[[5]](https://www.mdpi.com/2079-6374/13/8/781#:~:text=epinephrine%20takes%20place%20at%20a,anodic%20peak%20current%20increases%20linearly). (Oxidation peak ~+0.24 V, E_pa–E_pc ≈0.44 V↔–0.20 V). On bare electrodes, peaks ~0.3–0.35 V[[6]](https://www.sciencedirect.com/science/article/abs/pii/S1572665710003875#:~:text=Simultaneous%20determination%20of%20epinephrine%20and,EP%20and%20UA%20by). | ~0.20 V *[est. via catecholamine trends]*[[4]](https://pmc.ncbi.nlm.nih.gov/articles/PMC8822484/#:~:text=,For). *(Similar to other catecholamines at pH7.4)*. |
| **Serotonin (5-HT)** | ~0.40 V *[CV; glassy C]*[[7]](https://pubs.acs.org/doi/pdf/10.1021/ac9908748#:~:text=Electrochemical%20Oxidation%20of%20Histamine%20and,that%20for%20the%20diamond%20electrode). (Oxidation peak ~+0.4 V on GC; notably higher on inert surfaces, ~+0.6–0.7 V[[8]](https://www.sciencedirect.com/science/article/abs/pii/S1388248117301777#:~:text=Electrochemical%20detection%20of%20serotonin%20in,flat%20ITO%20and%20an).) | *No specific data.* (Likely moderate; e.g. phenolic indole with amine predicted in ~0.5–0.7 V range vs Ag/AgCl by DFT/ML trends[[9]](https://pmc.ncbi.nlm.nih.gov/articles/PMC12225624/#:~:text=donating%20functional%20groups%2C%20such%20as,KCl%29%29%20has%20a).) |
| **L-DOPA** (Levodopa) | ~0.55 V *[CV; glassy C]*[[10]](https://www.researchgate.net/publication/392688884_The_Influence_of_pH_on_the_Catalytic_Capacity_of_Levodopa_in_the_Electroreduction_Processes_of_Zn_Ions#:~:text=,It%20is%20also%20necessary). (Reversible couple E_pa≈+0.58, E_pc≈+0.52 V vs Ag/AgCl in PBS; formal E°′ ≈+0.55 V.) | ~0.13 V *[DFT-calculated; vs SHE→Ag/AgCl]*[[4]](https://pmc.ncbi.nlm.nih.gov/articles/PMC8822484/#:~:text=,The%20same%20calculations). *Standard E° ≈0.745 V vs SHE (pH0) implies ~0.33 V vs SHE at pH7.4 ≈0.13 V vs Ag/AgCl*. |
| **DOPAC** (3,4-Dihydroxyphenylacetic acid) | ~0.15–0.20 V *[CV; glassy C]* (Comparable to dopamine; similar catechol electrochemistry[[11]](https://pmc.ncbi.nlm.nih.gov/articles/PMC8871592/#:~:text=NIH%20pmc,build%20up%20to%20higher).) | ~0.25 V *[est. via functional group effect]*[[9]](https://pmc.ncbi.nlm.nih.gov/articles/PMC12225624/#:~:text=donating%20functional%20groups%2C%20such%20as,KCl%29%29%20has%20a). *(Electron-withdrawing –COO⁻ raises E°′ above unsubstituted catechols.)* |
| **Histamine** | ~1.10 V *[CV; carbon]*[[12]](https://pmc.ncbi.nlm.nih.gov/articles/PMC6777000/#:~:text=Mechanism%20of%20Histamine%20Oxidation%20and,carbon%20electrodes%2C%20including%20GCE%2C). (Requires ≥+1.1 V vs Ag/AgCl for oxidation at carbon; prone to electrode fouling via polymerization.) | ~1.0–1.2 V *[est. via DFT/ML trends]*[[9]](https://pmc.ncbi.nlm.nih.gov/articles/PMC12225624/#:~:text=donating%20functional%20groups%2C%20such%20as,KCl%29%29%20has%20a). *(Multiple amine groups; predicted E°′ higher than aniline (~0.71 V) due to aromatic imidazole.)* |

**Notes:** E°′ = formal (quasi-equilibrium) potential at pH ~7.4. Experimental values are from cyclic voltammetry (CV) in physiological buffer (PBS ~0.1 M) at 25 °C, using Ag/AgCl (sat’d KCl) reference electrodes. Predicted values are from theoretical calculations or machine-learning (ML) models, converted to the Ag/AgCl scale. Each entry lists representative peak or half-wave potentials and the measurement method or source. Sources are given in brackets.

**References**

[1] Kim D-S, Kang E-S, Baek S, Choo S-S, Chung Y-H, Lee D, Min J, Kim T-H. Electrochemical detection of dopamine using periodic cylindrical gold nanoelectrode arrays. Sci Rep. 2018;8;14049.

[2] Liu T, Han L-L, Du C-M, Yu Z-Y. Redox potentials of dopamine and its supramolecular complex with aspartic acid. Russ J Phys Chem A. 2014;88;1085–1090.

[3] Dăscălescu D, Apetrei C. Nanomaterials based electrochemical sensors for serotonin detection: A review. Chemosensors. 2021;9;14.

[4] Jodko-Piórecka K, Sikora B, Kluzek M, Przybylski P, Litwinienko G. Antiradical activity of dopamine, L-DOPA, adrenaline, and noradrenaline in water/methanol and in liposomal systems. J Org Chem. 2022;87;1791–1804.

[5] Leau SA, Lete C, Matei C, Lupu S. Electrochemical sensing platform based on metal nanoparticles for epinephrine and serotonin. Biosensors. 2023;13;781.

[6] Mazloum-Ardakani M, Beitollahi H, Amini M, Mirjalili F. Simultaneous determination of epinephrine and uric acid at a gold electrode modified by a 2-(2,3-dihydroxyphenyl)-1,3-dithiane self-assembled monolayer. J Electroanal Chem. 2011;651;243–249.

[7] Sarada BV, Rao TN, Tryk DA, Fujishima A. Electrochemical oxidation of histamine and serotonin at highly boron-doped diamond electrodes. Anal Chem. 2000;72;1632–1638.

[8] Matuschek L, Göbel G, Lisdat F. Electrochemical detection of serotonin in the presence of 5-hydroxyindoleacetic acid and ascorbic acid by use of 3D ITO electrodes. Electrochem Commun. 2017;81;69–72.

[9] Sharma S, Kaminsky N, Radinsky K, Amirav L. Predicting oxidation potentials with DFT-driven machine learning. J Chem Inf Model. 2025;65;5345–5351.

[10] Nieszporek J, Pańczyk T. The influence of pH on the catalytic capacity of levodopa in the electroreduction processes of Zn²⁺ ions. Molecules. 2025;30;2590.

[11] Shao Z, Venton BJ. Different electrochemical behavior of cationic dopamine from anionic ascorbic acid and DOPAC at CNT yarn microelectrodes. J Electrochem Soc. 2022;169;026506**.**

[12] Puthongkham P, Lee ST, Venton BJ. Mechanism of histamine oxidation and electropolymerization at carbon electrodes. Anal Chem. 2019;91;8366–8373.

**Table S2. List of BGE composition in all reviewed articles**

| **Analytes** | **BGE** | | **pH** | | | **Electrode type** | **LOD** | | **Reference** | | |
| --- | --- | --- | --- | --- | --- | --- | --- | --- | --- | --- | --- |
|  |  | |  | | |  |  | |  | | |
| DA, | 10 mM lithium phosphate | | 6.1 | | | 25 µm platinum wire microelectrode (on-column, laser-etched cellulose acetate decoupler) | DA: 0.2 nM | | [57] | | |
| DA, CA | 10 mM phosphate | | 6.0 | | | Gold film electrode directly sputtered onto the capillary tip (on-capillary electrode, Au OCE, end-column); also Pt and Cu-modified OCEs tested | 0.5–1.2 µM (≈0.6–2.6 fmol) | | [69] | | |
| DA, NE, E, L-DOPA, CA | (a) 5 mM Na₂HPO₄ + 5 mM NaH₂PO₄ + 20 mM SDS; (b) 10 mM Na₂HPO₄ + 25 mM Na₂B₄O₇ + 10 mM SDS (borate complexation) | | 7.0 | | | 10 µm carbon fiber microelectrode (amperometric, end-column) | N/A, nM range for catechols | | [51] | | |
| NE, IP, CA | 10 mM NaH₂PO₄ + 10 mM Na₂HPO₄; or 15 mM NaH₂PO₄ + 5 mM Na₂B₄O₇ + 10 mM SDS | | 6.8 | | | 8 µm carbon fiber microelectrode, inserted 200 µm into detection capillary, with etched-joint decoupler (HF-thinned wall, porous-glass type) | NE: 2.2×10⁻⁸ M (45 amol), IP: 8.5×10⁻⁸ M (180 amol), CA: 2.3×10⁻⁸ M (32 amol), | | [85] | | |
| DA, DOPAC | 20 mM phosphate buffer with 2 mM EDTA | | 7.4 | | | 7 µm carbon fiber microelectrode (200–300 µm exposed length), integrated etched end-column decoupler (HF-thinned wall, ~10 µm thick) | DA: 5 nM (≈38 amol); 4-MC: 27 amol | | [86] | | |
| DA, NE, E, L-DOPA, | 5 mM Na₂HPO₄ + 5 mM NaH₂PO₄ + 20 mM SDS (micellar phosphate buffer) | | 7.0 | | | 10 µm carbon fiber working electrode nserted into the end of the detection capillary (end-column configuration) | Not reported | | [81] | | |
| 5-HT, DA, NE, E (IP as internal standard) | 160 mM phosphate buffer | | 5.9 | | | arbon fiber microdisc array electrode (~60 fibers, 6 µm diameter each) positioned at the capillary outlet (end-column amperometric detection, CE–AD, three-electrode configuration, SCE reference) | 5-HT: 2.98 × 10⁻¹⁰ M, DA: 4.42 × 10⁻¹⁰ M, NE: 6.00 × 10⁻¹⁰ M, E: 5.22 × 10⁻¹⁰ M | | [89] | | |
| LDME, L-DOPA, DA | 50 mM phosphate buffer | | 7.0 | | | Carbon disk electrode in a vertical wall-jet electrochemical cell (end-column amperometric detection, CE–AD, three-electrode configuration, +1.00 V vs. Ag/AgCl) | DA: 9.7 ng/mL, LDME: 14.6 ng/mL, L-DOPA: 98 ng/mL | | [94] | | |
| DA, 5-HT, OA, TA | 200 mM phosphate buffer (NaH₂PO₄) + 1 mM tetraborate | | 4.5 | | | Carbon-fiber microdisk electrode (~34.5 µm diameter) positioned at the capillary outlet (end-column fast-scan cyclic voltammetry detection, CE–FSCV, two-electrode configuration, −0.4 to +1.3 V waveform) | DA, 5-HT: ~1 nM | | [97] | | |
| DA, E | 20 mM phosphate buffer | | 6.0 | | | Polyhistidine chemically modified carbon electrode (carbon fiber bundle, wall-jet configuration, end-column amperometric detection, CE–AD, +0.2–0.3 V vs. SCE) | DA: 6 × 10⁻⁹ M, E: 8 × 10⁻⁹ M | | [98] | | |
| 5-HT | 0.01 M phosphate | | 8.0 | | | Carbon microdisk electrode (end-column, 50 µm i.d. capillary, no decoupler), conically shaped tip (~0.4 mm) | N/A | | [90] | | |
| DA, NE, E, 5-HT, DOPAC, | Na₂HPO₄–KH₂PO₄ buffer, ionic strength 0.05–0.16 M; optimal 0.16 M for transmitters (pH 6.5) and 0.05 M for metabolites (pH 7.0) | | 6.5–7.0 | | | 300 µm carbon disk electrode, wall-jet configuration, three-electrode system (C working, Pt auxiliary, Ag/AgCl reference) | 5-HT: 1.0×10⁻⁷ M; DA: 1.0×10⁻⁷ M; NE: 3.0×10⁻⁸ M; E: 1.0×10⁻⁷ M | | [92] | | |
| 5-HT | 0.20 M phosphate buffer (NaH₂PO₄–Na₂HPO₄) | | 8.0 | | | 300 µm carbon disk electrode, wall-jet configuration, three-electrode setup (Pt auxiliary, SCE reference), electrokinetic injection with micro-injector | 0.03–0.13 µM; linear 0.1–500 µM | | [93] | | |
| 5-HT | 10 mM phosphate buffer (PBS) + 20 mM SDS; sample stacking in 1 mM PBS | | 7.2 | | | 300 µm carbon disk electrode (end-column three-electrode cell; Pt auxiliary, Ag/AgCl reference) | 9.7 – 41.8 nM (≈ 2.0–9.7 ng mL⁻¹ | | [95] | | |
| DA, E, NE, CA | 20 mM phosphate buffer; | | 5.8 | | | Integrated **on-capillary tubular sol–gel carbon composite electrode** (CCE), 300 µm electrode length, wall thickness ≈ 50 µm | DA 0.7 µM (0.76 fmol), CA 1.2 µM (0.63 fmol); | | [73] | | |
| DA, E, NE, CA | 0.02 M phosphate | | 5.5 | | | Sol–gel carbon composite electrode (CCE), 150 µm diameter, wall-jet configuration, three-electrode cell (SCE reference, Pt auxiliary), anodically pre-treated at +1.8 V for 2 min | DA: 300 amol; NE: 350 amol; E: 270 amol; CA: 220 amol | | [99] | | |
| DA, E | 25 mM phosphate | | 6.5 | | | Sol–gel derived carbon composite electrode (CCE), wall-jet configuration, diameters 75–250 µm; Ag/AgCl reference, Pt auxiliary | E: 3×10⁻⁸ M (75 µm CCE); DA: similar | | [100] | | |
| DA, NE, E, 5-HT | 0.18 M phosphate | | 5.8 | | | **Pt/MWCNTs@Pdop-modified carbon disk electrode** (25 µm i.d., 50 µm gap; Ag/AgCl reference, Pt auxiliary; detection potential 1.0 V) | 5-HT = 3.0 × 10⁻¹⁰ M; DA = 4.9 × 10⁻¹⁰ M; NE = 9.2 × 10⁻¹⁰ M; E = 7.4 × 10⁻¹⁰ M | | [74] | | |
| DA, CA | 10 mM phosphate | | 6.0 | | | **BDD microelectrode,** CVD-grown on sharpened Pt wire (76 µm), insulated in polypropylene pipet tip; end-column CE–EC configuration | DA: 7.8×10⁻⁸ M (1.7 fmol); CA: 1.2×10⁻⁷ M (2.6 fmol) | | [102] | | |
| DA, CA | 10 mM phosphate | | 6.0 | | | **Dual-parallel on-capillary electrodes (OCEs)** sputter-coated Au or Pt films (0.3 µm Au, 0.06 µm Pt) on fused-silica capillary tips; end-column configuration; dual potentiostat control | Catecholamines: 0.4–1.2 µM; | | [109] | | |
| DA, CA | 25 mM phosphate/Tris buffer | | 6.5 | | | 25 µm Pt disk microelectrode, end-column amperometric detector (with and without fracture decoupler), Ag/AgCl reference, Pt auxiliary | Not explicitly quantified | | [112] | | |
| DA, NE, E, IP, CA | 0.1 M phosphate | | 5.0–6.9 | | | **Parallel-opposed dual-electrode detector:** one gold film on-capillary electrode (12.5 µm i.d.) and one gold disk electrode (90 µm), arranged face-to-face (~3 µm gap) enabling redox cycling (generator–collector mode) | 12 nM (4.2 amol) for DA; 29 nM (5.9 amol) for CA | | [117] | | |
| DA, NE, E | 0.20 M sodium phosphate or 0.15 M MES | | 5.4-5.6 | | | **Gold wire electrode** (115 µm) integrated in a fiber-optic connector (MT-type cell); end-column configuration with Ag/AgCl quasi-reference electrode; 3.5 µm Mylar spacer controlling capillary–electrode gap | 0.45 µM; linear range 0.5–500 µM; RSD: 3.7% (current), 0.5% (migration time) | | [118] | | |
| DA, NE, E | 0.1 M phosphate | | 5.0 | | | **Gold or copper disk microelectrodes (200 µm)** mounted in a handy mixing-joint detection cell (screw alignment, 25 µm i.d. capillary, adjustable 100 µm capillary–electrode gap); three-electrode setup with Ag/AgCl reference and Pt counter | Catecholamines: 23–44 nM (11–21 amol); | | [119] | | |
| DA, NE, E, CA | 10 mM phosphate + 15 mM borate + 10 mM SDS | | 7.0 | | | **Carbon film-based interdigitated ring-shaped array (IDRA) microelectrode**, pyrolyzed carbon film on quartz, 2.5 µm finger width, 1.3 µm gap; off-column wall-jet detection with Nafion tubing decoupler | NE: 80 nM; E: 0.23 µM; CA: 35 nM; DA: 81 nM | | [124] | | |
| DA | 25 mM phosphate buffer; | | 7.0 | | | **Interdigitated microarray electrode (IDA)** — 8 microband pairs (2 µm width, 2 µm gap), Au film; integrated in a **microfabricated subnanoliter wall-jet flow cell** with redox cycling | DA: 4 nM | | [125] | | |
| L-DOPA, DA in rat serum | | 50 mM phosphate | | 7.0 | **Carbon disk electrode**, wall-jet detection cell, Ag/AgCl (3 M KCl) reference, Pt counter; CE voltage 17 kV, +1.00 V detection potential | | | DME: 14.6 ng mL⁻¹ (≈ 0.07 µM); L-DOPA: 98 ng mL⁻¹ (≈ 0.5 µM); DA: 9.7 ng mL⁻¹ (≈ 0.06 µM) | | [142] |  |
| 5-HT | | 0.20 M phosphate buffer (NaH₂PO₄–Na₂HPO₄) | | 8.0 | 300 µm carbon disk electrode, wall-jet configuration (end-column CE–EC), Pt auxiliary, SCE reference, +0.9 V vs SCE | | | 0.03–0.13 µM | | [143] |  |
| DA, 5-HT in rat brain tissue punches | | 150 mM NaH₂PO₄ + 1 mM β-CD | | 4.0 | Carbon-fiber disk microelectrode (34.5 µm diameter), etched capillary (20–30 µm o.d.) for electrical decoupling, two-electrode configuration (Ag/AgCl reference | | | DA: 5 ± 3 nM, 5-HT: 10 ± 3 nM, Ade: 50 ± 20 nM | | [145] |  |
| Him (in individual rat peritoneal mast cells) | | 15.6 mM NaH₂PO₄ – 24.4 mM Na₂HPO₄ | | 7.0 | **Carbon fiber microdisk bundle electrode** (~30 fibers, 6 µm each), three-electrode end-capillary configuration (SCE reference, Pt auxiliary), detection potential +1.30 V | | | 96 fmol per cell (mean of nine cells) | | [151] |  |
| L-DOPA, Carbidopa | | 80 mM phosphate buffer (NaH₂PO₄/Na₂HPO₄) | | 7.0 | **Carbon disk electrode** (500 µm graphite pencil lead), wall-jet configuration, three-electrode CE–AD setup (Pt auxiliary, Ag/AgCl 3 M KCl reference) | | | L-DOPA: 0.6 mg mL⁻¹; Carbidopa: 0.3 mg mL⁻¹ | | [152] |  |
| L-DOPA | | 40 mM phosphate buffer (NaH₂PO₄–Na₂HPO₄) | | 5.3 | **Carbon disk electrode**, wall-jet configuration, three-electrode CE–AD setup (Pt auxiliary, Ag/AgCl reference), detection potential +0.95 V | | | L-DOPA: 0.38 µg mL⁻¹ (≈ 2.0 µM); | | [153] |  |

|  | | | | | | | |  |  | | | | | | | | | | | | | |  | | | | | |  | | | | | | | | | |  | | | | | | | | | | | |  |  |  |  |
| --- | --- | --- | --- | --- | --- | --- | --- | --- | --- | --- | --- | --- | --- | --- | --- | --- | --- | --- | --- | --- | --- | --- | --- | --- | --- | --- | --- | --- | --- | --- | --- | --- | --- | --- | --- | --- | --- | --- | --- | --- | --- | --- | --- | --- | --- | --- | --- | --- | --- | --- | --- | --- | --- | --- |
| DA, CA, DOPAC | | | | | 20 mM MES | | | | | | | | | 6.0 | | | | | | | | | | 10 µm carbon fiber microelectrode, end-column (no porous-glass junction) | | | | | | | | | DA 64 amol, CA 56 amol (S/N = 2) | | | | | | | | | | [62] | | | | | |  |  |  |  |  |  |
| DA, CA | | | | | 25 mM MES | | | | | | | | | 5.65 | | | | | | | | | | 11 µm carbon fiber microelectrode, end-column and optimized end-column (etched capillary, 2 µm i.d.) | | | | | | | | | DA 31 amol (best 10 amol), CA 24 amol (best 11 amol) | | | | | | | | | | [65] | | | | | |  |  |  |  |  |  |
| 5-HT, NE, E, DA, IP, DOPAC, | | | | | 25 mM MES | | | | | | | | | 5.7 | | | | | | | | | | Carbon fiber microelectrode (~5 µm diameter) inserted into the capillary outlet (end-column amperometric detection, CE–AD, +0.7 V vs. SSCE, two-electrode configuration) | | | | | | | | | 5-HT: 6 amol, NE: 11 amol | | | | | | | | | | [82] | | | | | |  |  |  |  |  |  |
| DA, 5-HT, NE, E, IP, L-DOPA | | | | | 25–30 mM MES | | | | | | | | | 5. 5-5.55 | | | | | | | | | | Carbon fiber microelectrode (~5 µm diameter) inserted into the capillary outlet (end-column amperometric detection, CE–AD, two-electrode configuration, +0.7 V vs. SSCE) | | | | | | | | | 5-HT 0.7 amol | | | | | | | | | | [83] | | | | | |  |  |  |  |  |  |
| DA, DOPAC, NE, E | | | | | 100 mM MES + 2% 1-propanol | | | | | | | | | 5.5 | | | | | | | | | | Carbon fiber microelectrode (~5 µm diameter) positioned at the capillary outlet (end-column amperometric detection, CE–AD, two-electrode configuration, +0.7 V vs. Ag/AgCl) | | | | | | | | | Not reported | | | | | | | | | | [84] | | | | | |  |  |  |  |  |  |
|  | | | | |  | | | | | | | | |  | | | | | | | | | |  | | | | | | | | |  | | | | | | | | | |  | | | | | |  |  |  |  |  |  |
| DA, NE, E, DOPAC | | | | | 25 mM MES | | | | | | | | | 5.65 | | | | | | | | | | Carbon-fiber microelectrode inserted into the capillary outlet (end-column amperometric detection, CE–AD, two-electrode configuration, +0.8 V vs. SSCE) | | | | | | | | | DA, NE: 0.13 fmol mg⁻¹ protein, E: 0.37 fmol mg⁻¹ proteinDOPAC: 0.11 fmol mg⁻¹ protein | | | | | | | | | | [139] | | | | | |  |  |  |  |  |  |
| DA, DOPAC (± NE, E, 5-HT tentatively) | | | | | 25 mM MES | | | | | | | | | 5.65 | | | | | | | | | | Carbon fiber microelectrode inserted into the capillary outlet (end-column amperometric detection, CE–AD, two-electrode configuration, +0.8 V vs. SSCE; porous-glass decoupler) | | | | | | | | | 300 zmol | | | | | | | | | | [134] | | | | | |  |  |  |  |  |  |
| DA, NE, E, CA | | | | | (a) 0.02 M MES (pH 6.05) for normal CE; (b) 0.01 M phosphate buffer (pH 6.98) + 0.01–0.025 M SDS for micellar CE (MECC) | | | | | | | | | 6.0–7.0 | | | | | | | | | | 10 µm carbon fiber microelectrode, end-column, with porous-glass decoupler (off-column) | | | | | | | | | Normal CE: 0.2–0.4 fmol; MECC: 17–19 fmol (catechol) | | | | | | | | | | [51] | | | | | |  |  |  |  |  |  |
| DA, CA, 5-HT, NE, E | | | | | 20–30 mM MES with 0–20% (v/v) 2-propanol | | | | | | | | | 6.0 | | | | | | | | | | 50 µm Pt disk electrode (end-column, wall-jet type thin-layer EC cell, PTFE-guided alignment) | | | | | | | | | DA 3.0 amol (23 nM), CA 5.2 amol (66 nM) | | | | | | | | | | [67] | | | | | |  |  |  |  |  |  |
|  | | | | |  | | | | | | | | |  | | | | | | | |  | | | | | |  | | | | | | | | | | |  | | | | | | | | | | |  |  |  |  |  |
| DA, E, CA | | | | | 20 mM MES | | | | | | | | | 5.7–6.0 | | | | | | | | 9 µm carbon fiber microelectrode, off-column detection using porous cellulose acetate (CA) joint decoupler | | | | | | DA: 12 fmol; E: 14 fmol; CA: 6 fmol | | | | | | | | | | | [55] | | | | | | | | | | |  |  |  |  |  |
| DA, CA | | | | | 25 mM MES, adjusted with NaOH | | | | | | | | | 5.65 | | | | | | | | 5 µm carbon fiber microelectrode, scanning amperometric detection (stepped from −0.2 to +1.0 V vs Ag/AgCl), inserted into etched capillary tip (~40 µm i.d.) | | | | | | ~174 fmol (peak 1) and 287 fmol (peak 2), total ≈ 460 fmol dopamine per cell | | | | | | | | | | | [87] | | | | | | | | | | |  |  |  |  |  |
| DA, E, CA | | | | | 25 mM MES, adjusted with NaOH | | | | | | | | | 5.65 | | | | | | | | 5 µm carbon fiber microelectrode, etched end-column cell (thin-layer configuration), scanning electrochemical detection (voltammetric mode) | | | | | | DA: 360 amol (2.3 µM); Epinephrine: 600 amol (3.9 µM); Catechol: 740 amol (6.6 µM) | | | | | | | | | [88] | | | | | | | | | |  |  |  |  |  |  |  |  |
| DA, NE, E | | | | | 50 mM MES, pH 6.1; | | | | | | | | | 6.1 | | | | | | | | Two-electrode configuration (working + electrophoretic ground as polarized pseudo-reference); Graphite, Pt, Cu, and Au/Hg electrodes (wall-jet configuration) | | | | | | CAs: 10⁻⁵ M; | | | | | | | | | [91] | | | | | | | | | |  |  |  |  |  |  |  |  |
| DA, NE, E | | | | | 30 mM MES | | | | | | | | | 5.7 | | | | | | | | **Boron-doped diamond (BDD) microline electrode**, 300 × 50 µm exposed area, end-column configuration, Ag/AgCl reference, Pt auxiliary | | | | | | DA: 20 nM; NE: 23 nM; E: 19 nM | | | | | | | | | [104] | | | | | | | | | |  |  |  |  |  |  |  |  |
| DA, IP (is) | | | | | 25 mM MES adjusted to pH 5.65 with 0.2 M NaOH | | | | | | | | | 5.65 | | | | | | | | **Gold microband array electrode**, 8 individually addressable microbands (10 µm width, 10 µm spacing, 1 mm length), Pt counter electrode | | | | | | DA: 13 µM | | | | | | | | | [120] | | | | | | | | | |  |  |  |  |  |  |  |  |
| DA, E, NE, IP ( internal standard) | | | | | 0.15 M MES adjusted to pH 5.57 | | | | | | | | | | 5.6 | | | | | | | | | **Parallel-opposed dual-electrode detector** combining an **on-capillary electrode** and a **disk electrode** (generator–collector configuration, redox cycling); Ag/AgCl quasi-reference, Pt counter | | | | | DA: 0.41 nM; E: 0.14 nM; NE: 0.16 nM | | | | | | | | | [127] | | | | | | | | | | |  |  |  |  |  |  |
| DA, E, NE, 5-HT | | | | | 150 mM boric acid buffer with 1 mM ascorbic acid | | | | | | | | | | | 10.33 | | | | | | | 300 µm carbon disk electrode (end-column three-electrode setup: Pt auxiliary, SCE reference) | | | | | | | Try: 68.3 nM; 5-HT: 31.3 nM; Tyr: 6.15 nM; DA: 25.2 nM; E: 28.0 nM; NE: 32.1 nM | | | | | | | | | [96] | | | | | | | | | | |  |  |  |  |  |
| DA, E, NE, DOPAC, | | | | | 0.040 M borate | | | | | | | | | | | 9.6 | | | | | | | 300–500 µm graphite or 1 mm glassy-carbon disk electrode coated with **melanin-type polymer** wall-jet amperometric detector, 100 µm gap | | | | | | | DA ≈ 0.9 µM, E ≈ 1.0 µM, NE ≈ 0.8 µM (≈ 41 fmol injected) | | | | | | | | | [76] | | | | | | | | | | |  |  |  |  |  |
| DA, E, NE, DOPA | | | | | 0.035 M boric acid buffer | | | | | | | | | | | 9.7 | | | | | | | **Carbon nanotube paste electrode (CNTPE)**; end-column configuration with Pt auxiliaryand Ag pseudo-reference; | | | | | | | DA: 1.8 µM; E: 2.1 µM; NE: 2.8 µM; AA: 3.3 µM; DOPAC: 13.3 µM (all at 0.4 V vs Ag) | | | | | | | | | [75] | | | | | | | | | | |  |  |  |  |  |
| DA, NE, E, DOPA | | | | | | 250 mM boric acid, adjusted to pH 8.8 with 1 M KOH | | | | | | | | | 8.8 | | | | | | | | **Boron-doped diamond (BDD) microelectrode,** CVD-grown on sharpened 76 µm Pt wire, 3–5 µm film thickness, end-column CE–AD cell with Ag/AgCl reference, Pt auxiliary | | | | | | 40–250 nM (mass LOD 120–370 amol; NE ≈ 52 nM; DA ≈ 44 nM; DOPEG ≈ 250 nM) | | | | | | | | | | | [103] | | | | | | | | | | | |  |  |  |
| NE in rat heart tissue (atria, ventricles, septum) | | | | | | 250 mM boric acid adjusted with KOH | | | | | | | | | 8.8 | | | | | | | | **Boron-doped diamond (BDD) microelectrode** (CVD film on Pt wire 76 µm, ~200 µm exposed; end-column CE–AD cell with Ag/AgCl reference, Pt auxiliary) | | | | | | 0.051 µM (CE-EC standard); mass LOD 0.05 pg; in tissue: 0.034 µg g⁻¹ (ventricles/septum), 0.22 µg g⁻¹ (atria) | | | | | | | | | | | [105] | | | | | | | | | | | |  |  |  |
| NNE in rat heart, spleen, and small intestine tissue | | | | | | 250 mM borate adjusted to pH 8.8 with KOH | | | | | | | | | 8.8 | | | | | | | | **Boron-doped diamond (BDD) microelectrode**, conically shaped (CVD-grown on sharpened 76 µm Pt wire), end-column CE-AD configuration with Ag/AgCl reference and Pt auxiliary electrode | | | | | | 68 ± 19 nM (≈ 0.066 pg NE injected) | | | | | | | | | | | [106] | | | | | | | | | | | |  |  |  |
| DA, E, NE | | | | | | 35 mM boric acid (pH 9.5) | | | | | | | | | | 9.5 | | | | | | | | | **Integrated on-capillary dual Pt wire electrodes** (generator: +0.95 V, detector: −0.3 V vs Ag/AgCl), separated by ~20 µm; end-column dual-electrode CE–AD configuration | | | | | | N/A | | | | | | | | | | [110] | | | | | | | | | | | |  |  |
| DA, NE, E | | | | | | | 40 mM borate | | | | | | | | | | 9.6 | | | | | Melanin-type polymer–modified carbon electrode (graphite disk, ~500 µm) in wall-jet amperometric detection (+700 mV vs Ag pseudo-reference) | | | | | | | | | | DA: 0.9 µM , E: 1.0 µM , NE: 0.8 µM | | | | | | | | | | [43] | | | | | | | | | | | | |
| DA, NE, E, DOPAC, AA | | | | | | | 35 mM borate buffer | | | | | | | | | | 9.7 | | | | | Glassy carbon electrode modified with CNT/PEI dispersion (end-column amperometric detection, ~50 µm gap) | | | | | | | | | | DA: 40 nM, DOPAC: 59 nM | | | | | | | | | | [72] | | | | | | | | | | | | |
| DA, E, NE, CA | | | | | | | 0.1 M CAPS | | | | | | | | | | 10.0 | | | | | 10 µm carbon fiber microelectrode, off-column (porous-glass decoupler) | | | | | | | | | | ≈5 pmol injected (catecholamines) | | | | | | | | | | [1] | | | | | | | | | | | | |
|  | | | | | | |  | | | | | | | | | |  | | | | |  | | | | | | | | | |  | | | | | | | | | |  | | | | | | | | | | | | |
| DA, CA | | | | | | | 50 mM TES with 2% 1-propanola | | | | | | | | | | 7.2 | | | | | 5 µm carbon fiber microelectrode, etched conical tip (~2.5 µm), end-column (etched capillary outlet, 770 nm i.d.) | | | | | | | | | | 340 zmol (DA, CA) | | | | | | | | | | [45] | | | | | | | | | | | | |
| DA, CA | | | | | | | 50 mM TES with 2% 1-propanol | | | | | | | | | | 7.2 | | | | | 5 µm carbon fiber microelectrode, flame-etched conical tip (≈2.5 µm), end-column; 770 nm i.d. capillary with 13 µm etched outlet | | | | | | | | | | Dopamine: 400 ± 100 zmol; Catechol: 410 ± 80 zmol | | | | | | | | | | [46] | | | | | | | | | | | | |
| DA, NE, IP (is) | | | | | | | 0.1 M lithium acetate + 0.5 mM Na₂EDTA | | | | | | | | | | 4.75 | | | | | 33 µm carbon fiber microelectrode (end-column, inserted into cast Nafion decoupler, 1 mm length, 90 µm i.d.) | | | | | | | | | | 3.1 ± 0.6 nM (DA); 3.2 ± 0.2 nM (NE); 2.8 ± 0.4 nM (IP); 3.2 ± 0.4 nM (DHBA) | | | | | | | | | | [56] | | | | | | | | | | | | |
| DA, NE, E | | | | | | | | | 20 mM sodium citrate (pH 2.5) | | | | | | | | | | 2.5 | | | | **Integrated on-capillary gold wire electrode (25 µm)** mounted perpendicular to flow, three-electrode CE–AD cell (Ag/AgCl reference, Pt auxiliary); | | | | | | | | | | DA: 0.12 µM (S/N = 2); | | | | | | | | | | [68] | | | | | | | | | | |  |
| DA, E | | | | | | | | | 25 mM histidine–MES | | | | | | | | | | 5.9 | | | | **Gold microelectrode** (100 µm wire, polished disk, integrated in Plexiglas™ detection cell; Ag/AgCl reference, Pt counter); end-column configuration without decoupler | | | | | | | | | | 0.3 µM | | | | | | | | | | [121] | | | | | | | | | | |  |
| E, NE, IP | | | | | | | | | 50 mM Tris–phosphate + 25 mM heptakis(2,6-di-O-methyl)-β-cyclodextrin (DM-β-CD) | | | | | | | | | | 2.5 | | | | **Microfabricated interdigitated platinum microband array (IDE)** — 8 bands (10 µm width, 10 µm spacing, 2 mm length) | | | | | | | | | | 5 µM | | | | | | | | | | [126] | | | | | | | | | | |  |
| DA, DOPAC | | | | | | | | | 50 mM TES buffer | | | | | | | | | | 7.4 | | | | **Carbon fiber microelectrode (33 µm);** fracture-type decoupler; on-line microdialysis–CE–EC interface with cellulose acetate isolation joint; Ag/AgCl | | | | | | | | | | DA, HQ, DOPAC: 10–25 µM (test standards) | | | | | | | | | | [141] | | | | | | | | | | |  |
| DA in single rat pheochromocytoma (PC12) cell | | | | | | | | | 10 mM Tris buffer adjusted to pH 6.2 with H₃PO₄ | | | | | | | | | | 6.2 | | | | Carbon fiber microelectrode (150–300 µm exposed, 8 µm O.D.) inserted into capillary outlet; two-electrode configuration vs. SCE; end-column configuration | | | | | | | | | | 0.29–1.28 fmol per cell (mean 0.61 ± 0.30 fmol) | | | | | | | | | | [144] | | | | | | | | | | |  |
| DA, L-DOPA, E, NE, 5-HT | | | | | | | | | 10 mM TES buffer + 30 mM SDS + 2% 1-propanol | | | | | | | | | | 7.1 | | | | **Carbon fiber microelectrode (5 µm diameter)** coated with **Nafion**, end-column configuration vs. Ag/AgCl reference; two-electrode system, CE potential ≈ 514–563 V/cm | | | | | | | | | | L-DOPA: 4 amol; Epinephrine: 20 amol; others within attomole range | | | | | | | | | | [147] | | | | | | | | | | |  |
| L-DOPA, DA, TA, OA, 5-HT, and their metabolites | | | | | | | | | 10 mM TES, 30 mM SDS, 2% 1-propanol | | | | | | | | | | 7.1 | | | | **5 µm carbon fiber microelectrode**, etched capillary (13 µm i.d.) for electrical decoupling, two-electrode CE–AD configuration vs Ag/AgCl reference electrode (+0.75 V) | | | | | | | | | | L-DOPA: 747 fmol/head; DA: 16 fmol/head;; 5-HT: 61 fmol/head | | | | | | | | | | [149] | | | | | | | | | | |  |
| NE, E, DA, L-DOPA | | | | | | | | | | | | 10 mM TES + 10 mM SDS | | | | | | | | | | 7.0 | | | | | **Carbon fiber microelectrode (5 µm diameter),** etched capillary tip (12–15 µm i.d.) for deviceless electrical decoupling, two-electrode amperometric detection at +0.65 V vs Ag/AgCl | | | | | | | | | | | Single-cell: NE ≈ 33 ± 14 fmol; E ≈ 25 ± 11 fmol; DA ≈ 0.34 fmol (after L-DOPA incubation); release experiment: NE 8 fmol, E 6 fmol | | | | | | | | | | [151] | | | | | |  |
|  | | | | | | | | | | | |  | | | | | | | | | |  | | | | |  | | | | | | | | | | |  | | | | | | | | | |  | | | | | |  |
|  | | | | | | | | | | | |  | | | | | | | | | |  | | | | |  | | | | | | | | | | |  | | | | | | | | | |  | | | | | |  |
|  | | | | | | | | | | | |  | | | | | | | | | |  | | | | |  | | | | | | | | | | |  | | | | | | | | | |  | | | | | |  |
|  | | | | | | | | | | | |  | | | | | | | | | |  | | | | |  | | | | | | | | | | |  | | | | | | | | | |  | | | | | |  |
|  | | | | | | | | | | | |  | | | | | | | | | |  | | | | |  | | | | | | | | | | |  | | | | | | | | | |  | | | | | |  |

| Neurotransmitter | Oxidation/Reduction Reaction | | | Ref. |
| --- | --- | --- | --- | --- |
| Dopamine (DA) | 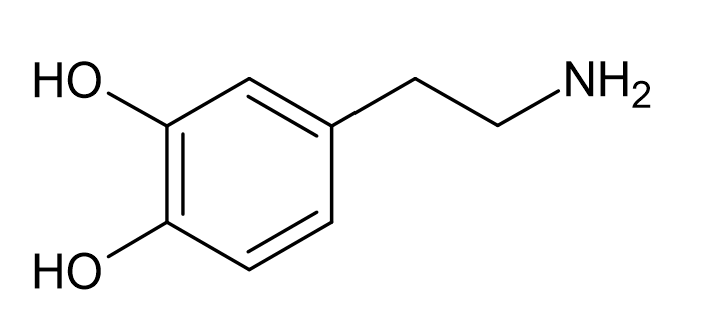 | 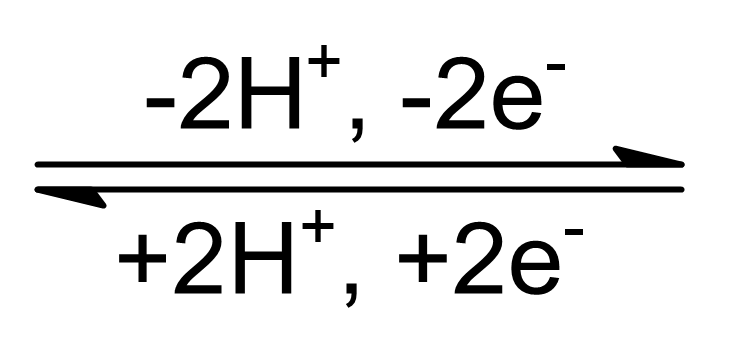 | 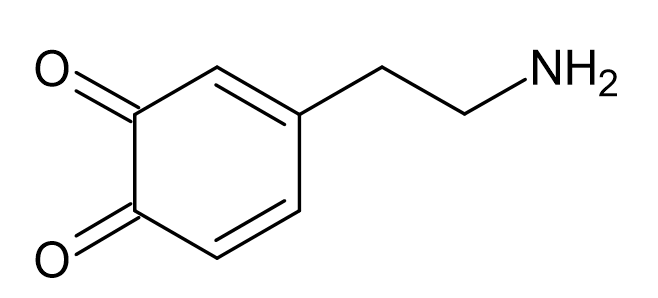 | [1] |
| Norepinephrine (NE) | 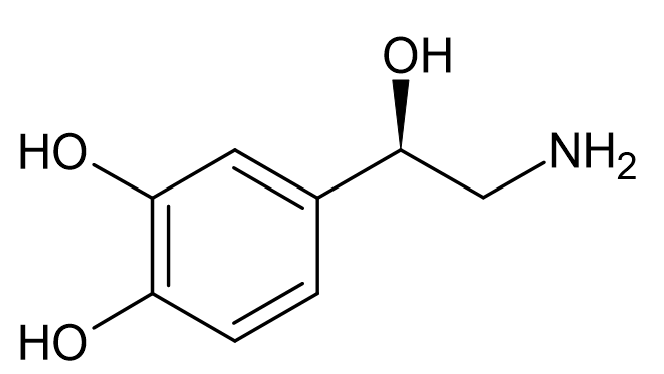 | 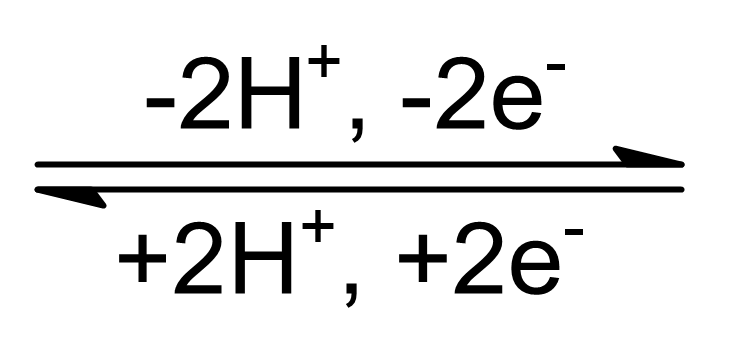 | 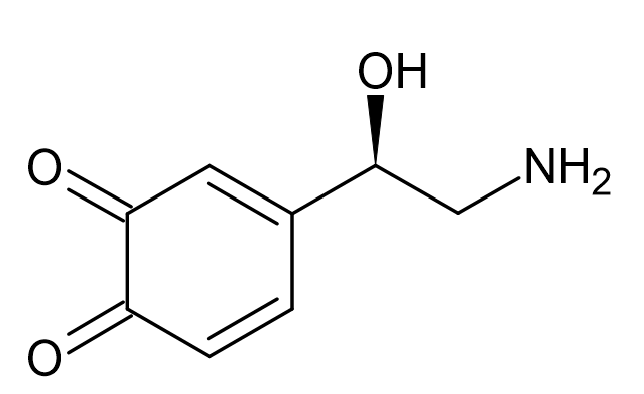 | [1] |
| Epinephrine (E) | 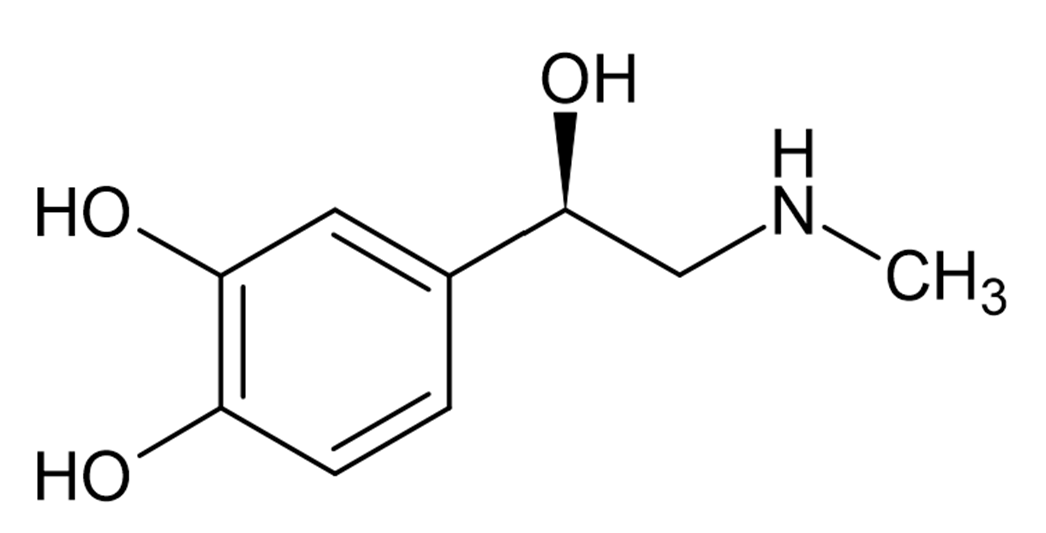 | 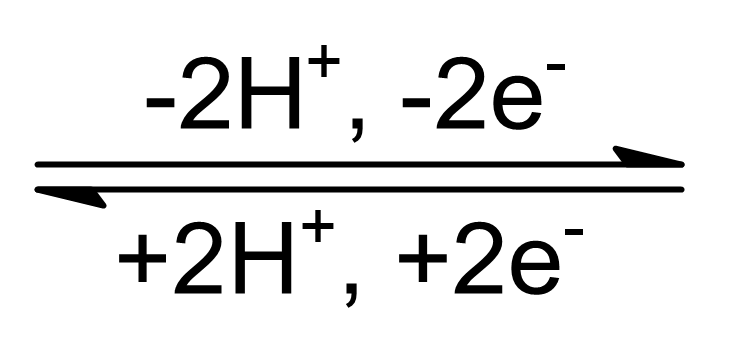 | 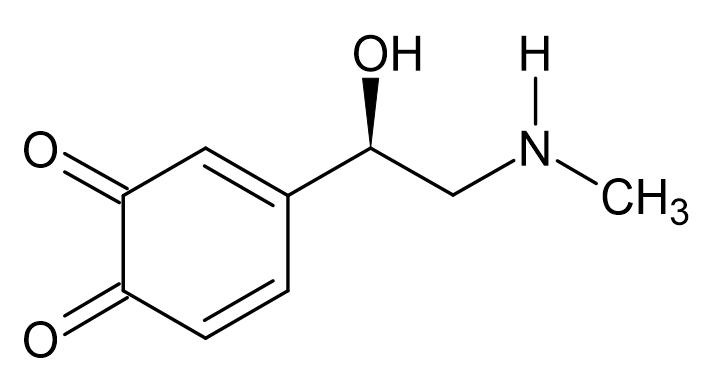 | [1] |
| Serotonin (5-HT) | 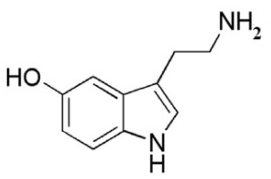 | 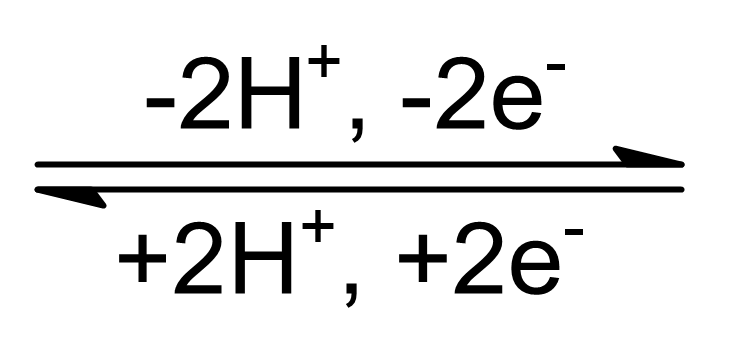 | 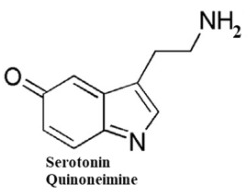 | [2] |
| Histamine (Him) | 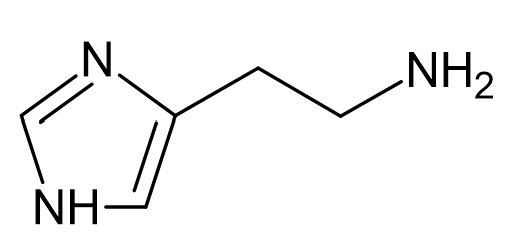 | 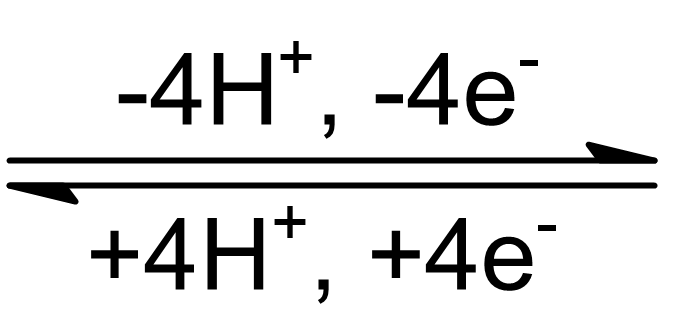 | 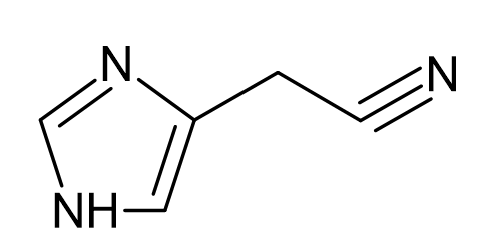 | [3, 4] |
| DOPAC | 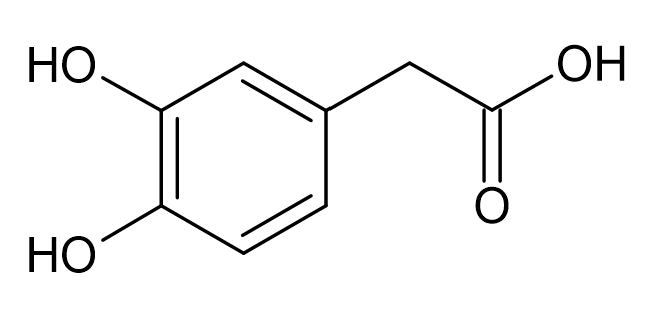 | 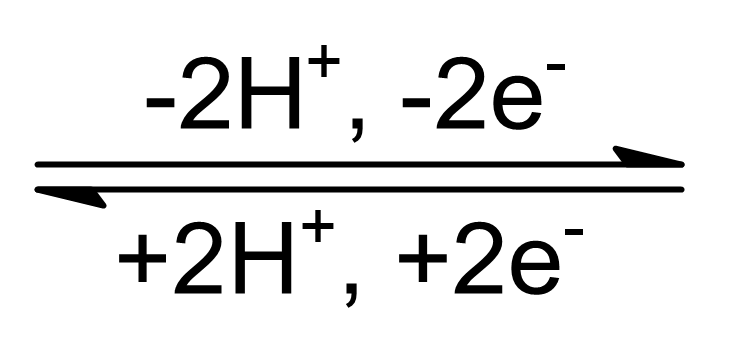 | 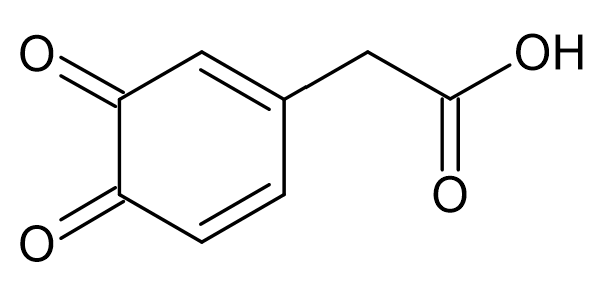 | [5, 6] |
| L-DOPA | 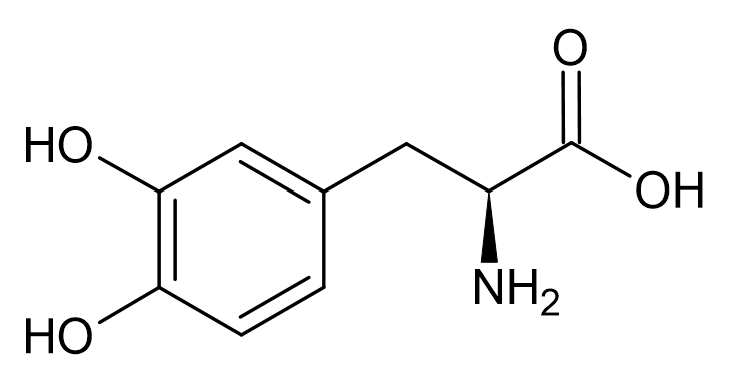 | 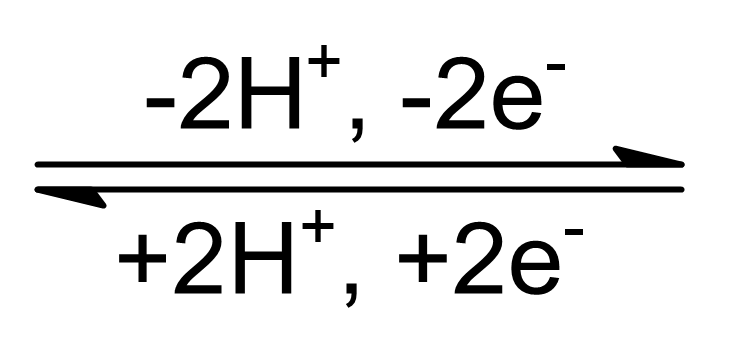 | 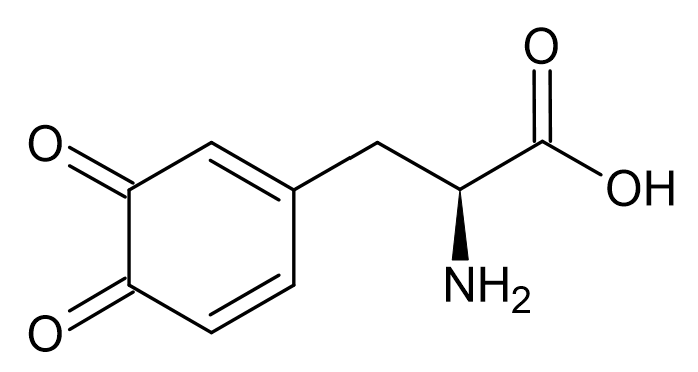 | [7] |

**Table S3. Oxidation/Reduction Reactions of Selected Neurotransmitters**

References:

[1] Hu M, Fritsch I. Application of electrochemical redox cycling: Toward differentiation of dopamine and norepinephrine. Anal Chem. 2016;88;5574–5578.

[2] Banu R, Kumara Swamy BE, Deepa S. Poly (fast sulphone black F) modified pencil graphite electrode sensor for serotonin. Sensors Int. 2020;1;100044.

[3]Degefu, H, Amare, M, Tessema, M, Admassie, S. Lignin modified glassy carbon electrode for the electrochemical determination of histamine in human urine and wine samples. Electrochim Acta. 2014;121;307–314.

[4] Anithaa AC, Mayil Vealan SB, Veerapandi G, Sekar C. Highly efficient non-enzymatic electrochemical determination of histamine based on tungsten trioxide nanoparticles for evaluation of food quality. J Appl Electrochem. 2021;51;1741–1753.

[5] Buleandră M, Popa DE, Gațe AT, Badea IA, Ciucu AA. Redox processes and detection of 3,4-dihydroxyphenylacetic acid at pencil graphite electrode. Microchem J. 2025;212;113544.

[6] Shao Z, Venton BJ. Different electrochemical behavior of cationic dopamine from anionic ascorbic acid and DOPAC at CNT yarn microelectrodes. J Electrochem Soc. 2022;169;026506.

[7] Santos AM, Wong A, Ferreira VS, Moraes FC, Fatibello-Filho O. Multivariate optimization of a novel electrode film architecture containing gold nanoparticle-decorated activated charcoal for voltammetric determination of levodopa levels in pre-therapeutic phase of Parkinson’s disease. Electrochim Acta. 2021;390;138851.
